# Association between TRP channels and glutamatergic synapse gene polymorphisms and migraine and the comorbidities anxiety and depression in a Chinese population

**DOI:** 10.3389/fgene.2023.1158028

**Published:** 2023-05-26

**Authors:** Mingxue Wang, Yujia Gu, Shuhan Meng, Lixin Kang, Jing Yang, Degang Sun, Yuxing Liu, Ze Wan, Yi Shan, Dongjie Xue, Chang Su, Shufen Li, Ran Yan, Yu Liu, Yashuang Zhao, Yonghui Pan

**Affiliations:** ^1^ Department of Epidemiology, School of Public Health, Harbin Medical University, Harbin, China; ^2^ Chronic Disease Prevention and Treatment Clinic, Heilongjiang Provincial Center for Disease Control and Prevention, Harbin, China; ^3^ Department of Neurology, Beidahuang Group Hongxinglong Hospital, Shuangyashan, China; ^4^ Catheterization Room, Beidahuang Group Hongxinglong Hospital, Shuangyashan, China; ^5^ Science and Education Section, Beidahuang Group Hongxinglong Hospital, Shuangyashan, China; ^6^ Physical Examination Section, Beidahuang Group Baoquanling Hospital, Hegang, China; ^7^ Department of Neurology, Hegang He Mine Hospital, Hegang, China; ^8^ Department of Internal Medicine, Baoquanling Farm Hospital, Hegang, China; ^9^ Vaccination Clinic, Baoquanling Farm Hospital, Hegang, China; ^10^ Department of Neurology, The First Affiliated Hospital of Harbin Medical University, Harbin, China

**Keywords:** migraine, gene polymorphisms, TRP channels, glutamate pathway, glutamatergic synapse

## Abstract

**Background:** Genetic and environmental factors contribute to migraine and the comorbidities of anxiety and depression. However, the association between genetic polymorphisms in the transient receptor potential (TRP) channels and glutamatergic synapse genes with the risk of migraine and the comorbidities of anxiety and depression remain unclear.

**Methods:** 251 migraine patients containing 49 comorbidities with anxiety and 112 with depression and 600 controls were recruited. A customized 48-plex SNPscan kit was used for genotyping 13 SNPs of nine target genes. Logistic regression was conducted to analyze these SNPs’ association with the susceptibility of migraine and comorbidities. The generalized multifactor dimension reduction (GMDR) was applied to analyze the SNP-SNP and gene-environment interactions. The GTEx database was used to examine the effects of the significant SNPs on gene expressions.

**Results:** The *TRPV1* rs8065080 and *TRPV3* rs7217270 were associated with an increased risk of migraine in the dominant model [OR_adj_ (95% CI): 1.75 (1.09–2.90), *p* = 0.025; 1.63 (1.02–2.58), *p* = 0.039, respectively]. *GRIK2* rs2227283 was associated with migraine in the edge of significance [OR_adj_ (95% CI) = 1.36 (0.99–1.89), *p* = 0.062]. In migraine patients, *TRPV1* rs222741 was associated with both anxiety risk and depression risk in the recessive model [OR_adj_ (95% CI): 2.64 (1.24–5.73), *p* = 0.012; 1.97 (1.02–3.85), *p* = 0.046, respectively]. *TRPM8* rs7577262 was associated with anxiety (OR_adj_ = 0.27, 95% CI = 0.10–0.76, *p* = 0.011). *TRPV4* rs3742037, *TRPM8* rs17862920 and *SLC17A8* rs11110359 were associated with depression in dominant model [OR_adj_ (95% CI): 2.03 (1.06–3.96), *p* = 0.035; 0.48 (0.23–0.96), *p* = 0.042; 0.42 (0.20–0.84), *p* = 0.016, respectively]. Significant eQTL and sQTL signals were observed for SNP rs8065080. Individuals with GRS (Genetic risk scores) of Q4 (14–17) had a higher risk of migraine and a lower risk of comorbidity anxiety than those with Genetic risk scores scores of Q1 (0–9) groups [OR_adj_ (95% CI): 2.31 (1.39–3.86), *p* = 0.001; 0.28 (0.08–0.88), *p* = 0.034, respectively].

**Conclusion:** This study suggests that *TRPV1* rs8065080, *TRPV3* rs7217270, and *GRIK2* rs2227283 polymorphism may associate with migraine risk. *TRPV1* rs222741 and *TRPM8* rs7577262 may associate with migraine comorbidity anxiety risk. rs222741, rs3742037, rs17862920, and rs11110359 may associate with migraine comorbidity depression risk. Higher GRS scores may increase migraine risk and decrease comorbidity anxiety risk.

## 1 Introduction

Migraine is a multifactorial neurovascular disorder in which genetic factors play a relevant role in both predisposing and determining underlying mechanisms. According to the Global Burden of Disease (GBD) Survey 2019, migraine ranked the second highest specific cause of disability worldwide and the first among women aged 15–49 years (Steiner et al., 2020).

Recently, the association of genetic, environmental, and migraine and its comorbidities of anxiety and depression have attracted wide attention (Louter et al., 2015). The comorbidity of depression and migraine may involve abnormal brain development, a common genetic basis, 5-hydroxytryptamine, sex hormones, and other mechanisms (Zhang et al., 2019; Yang et al., 2022; Lv et al., 2023). The calcitonin gene-related peptide (CGRP) is recognized as a critical player in the pathophysiology of migraine, which can trigger migraine attacks (Wattiez et al., 2020; Rees et al., 2022). Meanwhile, CGRP can act on the bed nucleus of the stria terminals (BNST) to evoke anxiety-like responses, activate anxiety-related structural neurons, and modulate behavioral responses to stress in rats (Sink et al., 2011). Given the common pathogenesis of anxiety, depression, and migraine, we hypothesize that there may be specific genetic characteristics sharing between migraine and the comorbidities of anxiety and depression.

Transient receptor potential (TRP) channels are a family of cation channels expressed primarily on the cell membrane. Activation of TRP channels is well-known to promote the release of CGRP from sensory nerve endings (Shibata and Tang, 2021). They have been used extensively as probes for the function of CGRP in various processes (Russell et al., 2014). Further, TRP channels have an essential role in migraine pain and associated symptoms, such as hyperalgesia and allodynia (Spekker et al., 2022). Several studies have explored the role of TRP channels gene polymorphisms in migraine across ethnic groups but with inconsistent results (Carreño et al., 2012; Chasman et al., 2014; Chen et al., 2018; Fu et al., 2019; Gavva et al., 2019; Yakubova et al., 2021; Siokas et al., 2022). Animal studies suggest that TRPA1 and TRPV1 antagonism might be a target for the treatment of anxiety and depression (de Moura et al., 2014; Escelsior et al., 2020; Ngoc et al., 2023). TRPA1 mRNA was colocalized with Edinger-Westphal nucleus (EWcp)/urocortin 1 (UCN1) neurons, and TRPA1 may contribute to the regulation of depression-like behavior and stress-adaptative responses in mice (Kormos et al., 2022). Moreover, it is well known that TRPV1 mediates glutamate in the brain (Fawley et al., 2014) and deficiency of TRPV1 induces antidepressant and anxiolytic effects by altering the expression of serotonin, gamma-aminobutyric acid (GABA) and glutamate *N*-methyl-d-aspartate (NMDA) receptors (You et al., 2012). The glutamate NMDA receptors play a relevant role in allodynia to mechanical stimuli, while TRPA1 channels may associate with NMDARs to promote ascending acute and chronic pain signals and to control mu-opioid receptors antinociception (Cortés-Montero et al., 2020). While the effects of TRPV1 on anxiety and depression may be related to cannabinoid 1 (CB1) receptor interactions (Sartim et al., 2017) and regulation of the glutamate/NMDA pathway (Lisboa and Guimarães, 2012).

In addition, malfunctions of the glutamatergic system may contribute to migraine symptoms (Gasparini et al., 2016). Glutamate can initiate migraine by cortical spreading depression (CSD), which is the lynchpin of migraine aura (Charles and Baca, 2013; Eikermann-Haerter, 2021). Thus far, only a few studies have focused on the relationship between the polymorphisms in ionotropic glutamate receptor genes (*GRIA1* and *GRIA3*) and migraine (Formicola et al., 2010; Maher et al., 2013; Fang et al., 2015). As the critical hub in neuronal metabolism, signaling, and plasticity, the role of glutamatergic synapse gene polymorphism in migraine is still not yet clarified (Frenguelli, 2022). Growing evidence supports the use of the NMDA receptor antagonist ketamine as a rapid-acting antidepressant, facilitating research into glutamate signaling modulators as depression treatment agents (Murrough et al., 2017; Hashimoto, 2019). However, the association of these genetic polymorphisms with migraine comorbidities anxiety, and depression remains unclear.

In summary, TRP is associated with hyperalgesia and allodynia, which mediates glutamate in the brain and can trigger migraine through CSD; moreover, activation of TRP channels can promote the release of CGRP, which can trigger migraine attacks. Therefore, we focused on candidate genes of TRP channels and glutamatergic synapses related to the proposed pathways mechanisms of migraine. This study aimed to examine the relationship between the polymorphisms of target 10 genes and migraine, migraine with or without aura, and anxiety and depression of migraine through a case control study in China.

## 2 Materials and methods

### 2.1 Study subjects

Subjects in this case control study were recruited from four hospitals (the First Affiliated Hospital of Harbin Medical University and three hospitals of Agricultural Reclamation, Heilongjiang Province: Hong Xinglong Administration Hospital, Bao Quanling Central Hospital, and Bao Quanling Farm Hospital) in northern China. The case group included 251 migraine patients, of which 49 patients were combined with anxiety and 112 were combined with depression. All the migraine patients were diagnosed according to the International Classification of Headache Disorders, 3rd edition, beta version (ICHD-3β version) criteria (Headache Classification Committee of the International Headache, 2013). The control group consisted of 600 nonmigraine subjects from the physical examination center of the same hospital in the same period. Considering the effect of age and especially gender differences on the results, we further conducted a sensitive analysis by matching the cases and controls at a 1:2 frequency based on sex concordance and age within 2 years. We conducted a questionnaire that included general basic information, lifestyle, medical history, anxiety, depression, and sleep status. All subjects signed informed consent prior to the study, and the study was approved by the Ethics Committee of Harbin Medical University.

### 2.2 Assessment of anxiety, depression, and sleep quality

Three self-assessment scales were used to assess anxiety, depression, and sleep quality, respectively. Self-Rating Anxiety Scale (SAS), which contains a 20-item questionnaire scored on a 4-point scale was used (Zung, 1971). The SAS scores were the raw score multiplied by 1.25. Then the SAS scores were divided into no anxiety (25–49 points), mild anxiety (50–60 points), moderate anxiety (61–70 points), and severe anxiety (71–100 points). The Patient Health Questionnaire-9 (PHQ-9) was used to assess depressive symptoms (Kroenke et al., 2001). PHQ-9 scores are divided into no depression (0–4 points), mild depression (5-9 points), moderate depression (10–14 points), and moderate to severe depression (15–27 points). The Pittsburgh Sleep Quality Index (PSQI) is composed of 19 items, which are classified into 7 components weighted from 0 to 3 (Buysse et al., 1989). PSQI score <5 indicated good sleep quality.

### 2.3 Candidate SNPs selection and genotyping

Based on the data from the previous literature and combined with the dbSNP database, 15 SNPs in 10 genes were identified (Table 1). The 8 tag SNPs, were selected through the dbSNPs database (http://www.ncbi.nlm.nih.gov/) with minor allele frequency (MAF) > 0.1 and *R* ^2^ > 0.8 in CHB (Han Chinese in Beijing) and CHS (Han Chinese in Southern). There is no significant linkage disequilibrium (*R* ^2^ < 0.8) among the candidate SNPs.

**TABLE 1:** Detailed information on 15 SNPs of TRP channels and glutamatergic synapse genes.

No.	SNPs	Chr	Position	Gene	Region	A1	A2	MAF	HWE	References
1	rs11110359	12	100774548	*SLC17A8*	synon_exon2	G	A	0.138	0.101	Tag SNP
2	rs11568537	12	100813976	*SLC17A8*	—	A	C	0.180	0.083	Tag SNP
3	rs2229900	6	34008006	*GRM4*	synon_exon8	A	G	0.435	0.030	Tag SNP
4	rs2227283	6	102503317	*GRIK2*	synon_exon15	G	A/T	0.347	0.404	Tag SNP
5	rs3761555	X	122316437	*GRIA3*	upstream	T	C	0.349	0.000	Formicola et al. (2010) Maher et al. (2013) Fang et al. (2015)
6	rs3745521	19	51170706	*SHANK1*	nonsynon_exon22	A	G	0.456	0.270	Tag SNP
7	rs3020047	11	70935766	*SHANK2*	5′-UTR_exon1	T	C	0.127	1.000	Tag SNP
8	rs55678639	11	70316391	*SHANK2*	3′-UTR_exon23	A	G	0.203	0.138	Tag SNP
9	rs8065080	17	3480447	*TRPV1*	nonsynon_exon13	T	C	0.394	0.616	Yakubova et al. (2021)
10	rs222741	17	3508880	*TRPV1*	intron2	G	A	0.193	0.270	Carreño et al. (2012)
11	rs7217270	17	3421475	*TRPV3*	intron15	A	G	0.063	0.254	Carreño et al. (2012)
12	rs3742037	12	110226379	*TRPV4*	synon_exon13	G	A	0.181	0.492	Tag SNP
13	rs17862920	2	234827995	*TRPM8*	intron1	C	T	0.250	0.927	Gavva et al. (2019)
14	rs10166942	2	234825093	near *TRPM8*	upstream	T	C	0.355	0.549	Chen et al. (2018) Siokas et al. (2022)
15	rs7577262	2	234818869	*TRPM8*	upstream	G	A	0.373	0.941	Chasman et al. (2014) Fu et al. (2019)

Abbreviations: Chr, Chromosome; A1, major allele; A2, minor allele; MAF, minor allele frequency; HWE, hardy weinberg equilibrium.

We collected 2 mL venous blood samples from all participants, separated out serum by centrifugation, and stored it at −80°C. The genomic DNA was extracted using the QIAamp^®^ DNA Blood Mini Kit (Qiagen, Germany). The extracted DNA was diluted to a working concentration of 30 ng/μl for further genotyping. The purity and concentration of DNA samples were measured by a Nanodrop 2000 spectrophotometer (Thermo Scientific, United States). Genotyping was performed using a customized 48-plex SNPscan kit (Gene Sky Biotechnologies Inc., Shanghai, China), which used double ligation and multiplex fluorescent PCR. We randomly chose 7% (58/851) of the samples and genotyped twice to ensure the accuracy and repeatability of genotyping, which were more than 95.0% (Supplementary Table S1).

### 2.4 Statistical analyses

The Hardy-Weinberg equilibrium (HWE) was analyzed by Fisher’s test using the GWASExactHW package of R 4.0.3 (Wigginton et al., 2005). We conducted logistic regression to examine the association between SNPs and the number of risk alleles and migraine. The gene-gene interaction analysis was conducted by GMDR software Beta version 0.7 (http://www.ssg.uab.edu/gmdr/). Power analysis using PASS 15 software. Statistical analyses were performed using statistical software R 4.0.3. *p* < 0.05 was considered statistically significant. Multiple testing adjustments were controlled by Bonferroni correction (0.05/13).

### 2.5 Bioinformatic analyses

To further examine the potential function of the significant SNPs on gene expression, we utilized the GTEx database (https://gtexportal.org/home/) to investigate the tissue-specific expression quantitative trait loci (eQTL) and splicing quantitative trait loci (sQTL). The Genotype-Tissue Expression (GTEx) project aims to collect and analyze multiple human tissues that are densely genotyped and analyzed for global RNA expression (Consortium, 2015). We also used the website (https://snpinfo.niehs.nih.gov/) to make functional predictions for significant SNPs (Xu and Taylor, 2009).

## 3 Results

### 3.1 Characteristics of the study subjects

The basic characteristics of all participants are summarized in Table 1; Supplementary Table S8, which include characteristics such as age, gender, lifestyle, and history of the disease. We recruited 600 controls and 251 migraine patients, of which 51 (20.32%) had migraine with aura (MA) and 200 (79.68%) had migraine without aura (MO). We observed that the migraine groups had a higher proportion of women, a higher proportion of anxiety, depression, and poor sleep quality (*p* = 0.001, *p* = 0.002, *p* < 0.001, *p* < 0.001, respectively) than the controls. Moreover, migraine patients had a lower proportion of alcohol consumption and exercise habits (*p* = 0.015, *p* = 0.001, respectively). Of the 251 migraines, 49 (19.52%) were combined with anxiety, and 112 (44.62%) were combined with depression. Among 851 participants, 281 cases were anxiety or depression including 123 (43.77%) in migraines and 158 (56.23%) in controls. Compared to the controls, the features of the MA and MO groups were almost the same as migraineurs. There were no significant differences between the MA and MO groups in terms of age and sex, but PHQ-9 scores (5.28 ± 4.66 vs. 3.64 ± 4.03, *p* = 0.024) and PSQI scores (5.77 ± 3.11 vs. 4.56 ± 2.72, *p* = 0.013) differed between the two groups (Table 2).

**TABLE 2:** Basic demographic and clinical characteristics of the migraine, MA, MO and control groups.

Characteristics	Control N = 600	Migraine N = 251	MA N = 51	MO N = 200	*P*_a_	*P*_b_	*P*_c_	*P*_d_
Age, Mean ± SD	43.12 ± 13.58	42.49 ± 14.11	41.69 ± 13.99	42.69 ± 14.17	0.544	0.483	0.705	0.649
Age grade (%)					0.936	0.894	0.968	0.936
≤35	254 (42.33)	109 (43.43)	24 (47.06)	85 (42.50)				
35∼	141 (23.50)	56 (22.31)	10 (19.611)	46 (23.00)				
50∼	127 (21.17)	56 (22.31)	11 (21.57)	45 (22.50)				
60∼	78 (13.00)	30 (11.95)	6 (11.77)	24 (12.00)				
Sex (%)					**0.001**	0.244	**0.001**	0.718
Male	195 (32.50)	52 (20.72)	12 (23.53)	40 (20.00)				
Female	405 (67.50)	199 (79.28)	39 (76.47)	160 (80.00)				
BMI (kg/m2), Mean ± SD	23.71 ± 3.46	23.06 ± 3.23	22.57 ± 2.81	23.19 ± 3.32	**0.009**	**0.008**	0.056	0.181
Marital status (%)					0.332	0.760	0.358	1.000
Unmarried	243 (40.50)	92 (36.65)	19 (37.26)	73 (36.50)				
Married	357 (59.50)	159 (63.35)	32 (62.75)	127 (63.50)				
Nationality (%)					0.605	0.164	1.000	0.206
Han nationality	554 (92.33)	235 (93.63)	50 (98.04)	185 (92.50)				
Minority	46 (7.67)	16 (6.38)	1 (1.96)	15 (7.50)				
Educational level (%)					**<0.001**	0.056	**<0.001**	0.369
Elementary and below	23 (3.83)	25 (9.96)	6 (11.77)	19 (9.50)				
Junior school	60 (10.00)	57 (22.71)	7 (13.73)	50 (25.00)				
Senior school	109 (18.17)	36 (14.34)	9 (17.65)	27 (13.50)				
College and above	408 (68.00)	133 (52.99)	29 (56.86)	104 (52.00)				
Smoking (%)					0.312	0.367	0.502	0.664
Yes	120 (20.00)	42 (16.73)	7 (13.73)	35 (17.50)				
No	480 (80.00)	209 (83.27)	44 (86.28)	165 (82.50)				
Alcohol (%)					**0.015**	0.230	**0.031**	1.000
Yes	145 (24.17)	41 (16.34)	8 (15.69)	33 (16.50)				
No	455 (75.83)	210 (83.67)	43 (84.31)	167 (83.50)				
Exercise (%)					**0.001**	0.161	**0.002**	0.949
Yes	303 (50.50)	95 (37.85)	20 (39.22)	75 (37.50)				
No	297 (49.50)	156 (62.15)	31 (60.78)	125 (62.50)				
History of hypertension (%)					0.363	0.120	0.773	0.308
Yes	51 (8.50)	27 (10.76)	8 (15.69)	19 (9.50)				
No	549 (91.50)	224 (89.24)	43 (84.31)	181 (90.50)				
History of diabetes (%)					0.107	0.511	0.190	1.000
Yes	34 (5.67)	7 (2.79)	1 (1.961)	6 (3.00)				
No	566 (94.33)	244 (97.21)	50 (98.04)	194 (97.00)				
Family history of diabetes (%)					0.094	0.182	0.205	0.657
Yes	92 (15.33)	51 (20.32)	12 (23.53)	39 (19.50)				
No	508 (84.67)	200 (79.68)	39 (76.47)	161 (80.50)				
Family history of migraine (%)					**<0.001**	**0.048**	**<0.001**	0.903
Yes	113 (18.83)	83 (33.07)	16 (31.37)	67 (33.50)				
No	487 (81.17)	168 (66.93)	35 (68.63)	133 (66.50)				
SAS score, Mean ± SD	36.83 ± 9.79	36.23 ± 11.50	38.77 ± 13.42	35.59 ± 10.90	0.475	0.317	0.155	0.122
SAS grade (%)					**0.002**	**0.001**	0.052	0.088
No anxiety	537 (89.50)	202 (80.48)	36 (70.59)	166 (83.00)				
Mild anxiety	53 (8.83)	41 (16.34)	12 (23.53)	29 (14.50)				
Moderate anxiety	10 (1.67)	8 (3.19)	3 (5.88)	5 (2.50)				
PHQ-9 score, Mean ± SD	2.73 ± 3.87	3.97 ± 4.21	5.28 ± 4.66	3.64 ± 4.03	**<0.001**	**<0.001**	**0.006**	**0.024**
PHQ-9 grade (%)					**<0.001**	**<0.001**	**<0.001**	0.100
No depression	457 (76.17)	139 (55.38)	21 (41.18)	118 (59.00)				
Mild depression	87 (14.50)	78 (31.08)	20 (39.22)	58 (29.00)				
Moderate depression	42 (7.00)	22 (8.77)	6 (11.77)	16 (8.00)				
Moderate to severe depression	14 (2.33)	12 (4.78)	4 (7.84)	8 (4.00)				
PSQI score, Mean ± SD	3.82 ± 2.60	4.801 ± 2.84	5.77 ± 3.11	4.56 ± 2.72	**<0.001**	**<0.001**	**0.001**	**0.013**
PSQI grade (%)					**<0.001**	**0.001**	**0.002**	0.157
Good sleep quality	397 (66.17)	128 (51.00)	21 (41.18)	107 (53.50)				
Poor sleep quality	203 (33.83)	123 (49.00)	30 (58.82)	93 (46.50)				

Abbreviations: MA, migraine with aura; MO, migraine without aura; BMI, body mass index; SAS, the Self-Rating Anxiety Scale; PHQ-9, the Patient Health Questionnaire-9; PSQI, the Pittsburgh Sleep Quality Index. *p*_a_, comparison of Migraine with Controls; *p*_b_, comparison of MA with Controls; *p*_c_, comparison of MO with Controls; *p*_d_, comparison of MA with MO. *p* < 0.05 are considered statistically significant, and shown in bold.

### 3.2 Association between gene polymorphisms and migraine susceptibility

The results showed that except for the *GRM4*-rs2229900 and *GRIA3*-rs3761555 loci which did not conform to the HWE (*p* < 0.05), the remaining 13 SNPs were all agreed with the HWE (*p* > 0.05) and were included in the subsequent analysis (Table 1).

As shown in Table 3, *TRPV1* rs8065080 was associated with an increased risk of migraine in the dominant model (CC + TC vs. TT: OR_adj_ = 1.78, 95% CI = 1.09–2.90, *p* = 0.025). *TRPV1* rs8065080 genotype TT decreased migraine risk compared to wild type CC genotype (OR_adj_ = 0.55, 95% CI = 0.32–0.92, *p* = 0.025). *TRPV3* rs7217270 was associated with increased migraine risk based on the dominant model (AA + AG vs. GG: OR_adj_ = 1.63, 95% CI = 1.02–2.58, *p* = 0.039). However, none of the associations was significant after Bonferroni correction for multiple tests with *p* < 0.0038 (0.05/13). *GRIK2* rs2227283 was associated with migraine in the edge of significance [OR_adj_ (95% CI) = 1.36 (0.99–1.89), *p* = 0.062]. In univariate analysis, *TRPM8* rs7577262 and *TRPM8* rs17862920 were associated with migraine [OR (95% CI): 0.68 (0.49–0.93), *p* = 0.016; 0.71 (0.52–0.97), *p* = 0.031, respectively]. However, after adjusting for confounders these associations narrowly avoided significance with *p* of 0.120 and 0.141. Additional nonsignificant results are in the Appendix (Supplementary Table S2).

**TABLE 3:** Associations between the *GRIK2, TRPV1*, *TRPV3* and *TRPM8* gene polymorphisms and the risk of migraine.

SNPs/genotypes/genetic models	Control No. (%)	Migraine No. (%)	OR (95% CI)	*p*	OR_adj_ (95% CI)	*p*
*GRIK2*-rs2227283 G>A						
GG	269 (44.83)	103 (41.04)	ref		ref	
GA	249 (41.50)	124 (49.40)	1.30 (0.95–1.78)	0.100	1.28 (0.91–1.80)	0.162
AA	82 (13.67)	24 (9.56)	0.76 (0.45–1.26)	0.300	0.74 (0.42–1.26)	0.277
Dominant model			1.50 (0.94–2.47)	0.100	1.54 (0.93–2.65)	0.105
Recessive model			1.38 (1.02–1.85)	**0.034**	1.36 (0.99–1.89)	0.062
Additive model			0.73 (0.54–0.98)	**0.034**	0.73 (0.53–1.02)	0.062
*TRPV1*-rs8065080 C>T						
CC	212 (35.33)	95 (37.85)	ref		ref	
TC	287 (47.83)	128 (51.00)	1.00 (0.72–1.37)	0.977	0.92 (0.65–1.31)	0.650
TT	101 (16.83)	28 (11.16)	0.62 (0.38–0.99)	0.052	0.55 (0.32–0.92)	**0.025**
Dominant model			1.61 (1.04–2.56)	**0.036**	1.75 (1.09–2.90)	**0.025**
Recessive model			1.14 (0.85–1.53)	0.400	1.09 (0.79–1.50)	0.603
Additive model			0.88 (0.66–1.18)	0.400	0.92 (0.67–1.27)	0.603
*TRPV3*-rs7217270 G>A						
GG	535 (89.17)	210 (83.67)	ref		ref	
AG	62 (10.33)	38 (15.14)	1.56 (1.01–2.40)	**0.044**	1.59 (0.98–2.55)	0.056
AA	3 (0.50)	3 (1.20)	2.55 (0.47–13.86)	0.254	2.28 (0.39–13.39)	0.340
Dominant model			1.61 (1.05–2.44)	**0.028**	1.63 (1.02–2.58)	**0.039**
Recessive model			2.40 (0.32–18.07)	**0.368**	1.58 (0.98–2.53)	0.060
Additive model			0.65 (0.99–0.42)	**0.047**	0.63 (0.40–1.03)	0.060
*TRPM8*-rs7577262 G>A						
GG	220 (36.67)	116 (46.22)	ref		ref	
GA	294 (49.00)	105 (41.83)	0.68 (0.49–0.93)	**0.016**	0.76 (0.54–1.07)	0.120
AA	86 (14.33)	30 (11.95)	0.66 (0.41–1.05)	0.087	0.70 (0.41–1.15)	0.166
Dominant model			1.23 (0.80–1.95)	0.357	1.25 (0.78–2.06)	0.366
Recessive model			0.75 (0.56–1.01)	0.056	0.83 (0.60–1.15)	0.258
Additive model			1.34 (0.99–1.80)	0.056	1.21 (0.87–1.67)	0.258
*TRPM8*-rs17862920 C>T						
CC	321 (53.50)	157 (62.55)	ref		ref	
CT	237 (39.50)	82 (32.67)	0.71 (0.52–0.97)	**0.031**	0.77 (0.54–1.09)	0.141
TT	42 (7.00)	12 (4.78)	0.58 (0.29–1.11)	0.115	0.66 (0.30–1.33)	0.258
Dominant model			1.50 (0.80–3.02)	0.229	1.38 (0.69–2.95)	0.383
Recessive model			0.74 (0.54–1.01)	0.061	0.80 (0.57–1.12)	0.203
Additive model			1.35 (0.99–1.84)	0.061	1.25 (0.89–1.76)	0.203

Bold type indicates *p* < 0.05. OR_adj_ (95% CI) adjusted factors: age, sex, BMI, marital status, nationality, educational level, smoking, alcohol, exercise, history of hypertension, history of diabetes, family history of diabetes, family history of migraine, SAS grade, PHQ9 grade, PSQI grade.

*GRIK2*, glutamate ionotropic receptor kainate type subunit 2; *TRPV1*, transient receptor potential cation channel subfamily V member 1; *TRPV3*, transient receptor potential cation channel subfamily V member 3; *TRPM8*, transient receptor potential cation channel subfamily M member 8.

Furthermore, we conducted a sensitivity analysis by matching the cases and controls at a 1:2 frequency based on sex and age within 2 years. Consistently, we observed only three of the five SNPs (*TRPV3* rs7217270, *TRPM8* rs7577262, and *TRPM8* rs17862920) in association with migraine and in the same direction as the unmatched data results, but possibly marginally significant after multifactorial adjustment with *p* of 0.058, 0.110 and 0.105, respectively. Differently, the associations of *TRPV1* rs8065080 and *GRIK2* rs2227283 with migraine were not significant due to the small sample size in matched data (Supplementary Table S8).

### 3.3 Subgroup analysis

In addition, the results of subgroup analysis showed that the frequency of GA genotype of *SLC17A8* rs11110359 was marked higher in the MO group compared to the control (OR_adj_ = 1.56, 95% CI = 1.03–2.34, *p* = 0.034) and showed a significant risk for MO patients in three models (Dominant: OR_adj_ = 1.51, 95% CI = 1.02–2.24, *p* = 0.040; Recessive: OR_adj_ = 1.55, 95% CI = 1.03–2.33, *p* = 0.036; Additive: OR_adj_ = 0.65, 95% CI = 0.43–0.98, *p* = 0.036, respectively). The CC genotype of *SLC17A8* rs11568537 was associated with decreased MO risk compared to the AA genotype (OR_adj_ = 0.22, 95% CI = 0.05–0.70, *p* = 0.021) and increased MO risk in the dominant model (OR_adj_ = 4.40, 95% CI = 1.42–19.57, *p* = 0.023). The genotype TT of *TRPV1* rs8065080 was associated with decreased MO risk (OR_adj_ = 0.55, 95% CI = 0.31–0.97, *p* = 0.043). rs8065080 increased MO risk in the dominant model (CC + TC vs. TT: OR_adj_ = 1.82, 95% CI = 1.09–3.17, *p* = 0.023). *TRPV3* rs7217270 and *TRPM8* rs17862920 were associated with MO risk in a very slight trend toward significance with *p* of 0.056 and 0.169. *SHANK1* rs3745521 and *TRPM8* rs7577262 were associated with MA risk and tended to approach significance with *p* of 0.070 and 0.081 (Table 4). However, all the *p*-values did not withstand Bonferroni correction.

**TABLE 4:** Associations between *SLC17A8*, *SHANK1, TRPV1, TRPV3* and *TRPM8* gene polymorphisms and the risk of migraine by aura.

SNPs/genotypes/genetic models	Control No. (%)	MA					MO				
		No. (%)	OR (95% CI)	*p*	OR_adj_ (95% CI)	*p*	No. (%)	OR (95% CI)	*p*	OR_adj_ (95% CI)	*p*
*SLC17A8*-rs11110359 G>A											
GG	462 (77.00)	42 (82.35)	ref		ref		141 (70.50)	ref		ref	
GA	122 (20.33)	9 (17.65)	0.81 (0.36–1.64)	0.584	0.81 (0.34–1.744)	0.604	54 (27.00)	1.45 (1.00–2.10)	0.050	1.56 (1.03–2.34)	**0.034**
AA	16 (2.67)	0 (0.00)	—	—	—	—	5 (2.50)	1.02 (0.33–2.67)	0.964	1.17 (0.35–3.28)	0.785
Dominant model			0.72 (0.32–1.45)	0.382	0.72 (0.30–1.538)	0.416		1.40 (0.98–2.00)	0.065	1.51 (1.02–2.24)	**0.040**
Recessive model			0.84 (0.37–1.70)	0.646	0.84 (0.35–1.808)	0.669		1.45 (1.00–2.09)	**0.049**	1.55 (1.03–2.33)	**0.036**
Additive model			1.19 (0.59–2.67)	0.646	1.19 (0.55–2.837)	0.669		0.69 (0.48–1.00)	**0.049**	0.65 (0.43–0.98)	**0.036**
*SLC17A8*-rs11568537 A>C											
AA	404 (67.33)	31 (60.78)	ref		ref		142 (71.00)	ref		ref	
AC	167 (27.83)	16 (31.37)	1.25 (0.65–2.31)	0.490	1.29 (0.63–2.556)	0.479	55 (27.50)	0.94 (0.65–1.34)	0.723	0.94 (0.63–1.37)	0.737
CC	29 (4.83)	4 (7.84)	1.80 (0.51–4.94)	0.299	1.72 (0.43–5.475)	0.396	3 (1.50)	0.29 (0.07–0.85)	**0.046**	0.22 (0.05–0.70)	**0.021**
Dominant model			0.60 (0.22–2.07)	0.352	0.63 (0.20–2.463)	0.457		3.34 (1.17–14.03)	**0.049**	4.40 (1.42–19.57)	**0.023**
Recessive model			1.19 (0.62–2.16)	0.590	1.23 (0.61–2.411)	0.554		0.98 (0.68–1.40)	0.927	1.00 (0.67–1.46)	0.978
Additive model			0.84 (0.46–1.60)	0.590	0.81 (0.42–1.652)	0.554		1.02 (0.71–1.46)	0.927	1.01 (0.69–1.49)	0.978
*SHANK1*-rs3745521 A>G											
AA	173 (28.83)	12 (23.53)	ref		ref		68 (34.00)	ref		ref	
AG	293 (48.83)	21 (41.18)	1.03 (0.50–2.22)	0.930	0.93 (0.42–2.12)	0.857	93 (46.50)	0.81 (0.56–1.17)	0.251	0.70 (0.47–1.05)	0.083
GG	134 (22.33)	18 (35.29)	1.94 (0.91–4.26)	0.090	1.78 (0.77–4.24)	0.184	39 (19.50)	0.74 (0.47–1.16)	0.194	0.63 (0.38–1.03)	0.067
Dominant model			0.53 (0.29–0.98	**0.038**	0.54 (0.28–1.07)	0.070		1.19 (0.80–1.79)	0.400	1.28 (0.84–1.98)	0.267
Recessive model			0.73 (0.41–1.30)	0.295	0.68 (0.36–1.28)	0.241		0.91 (0.66–1.26)	0.567	0.85 (0.60–1.20)	0.361
Additive model			1.36 (0.77–2.47)	0.295	1.46 (0.78–2.79)	0.241		1.10 (0.80–1.52)	0.567	1.18 (0.83–1.66)	0.361
*TRPV1*-rs8065080 C>T											
CC	212 (35.33)	22 (43.14)	ref		ref		73 (36.50)	ref		ref	
TC	287 (47.83)	23 (45.10)	0.77 (0.42–1.43)	0.407	0.64 (0.32–1.27)	0.204	105 (52.50)	1.06 (0.75–1.51)	0.732	1.01 (0.69–1.47)	0.962
TT	101 (16.83)	6 (11.77)	0.57 (0.21–1.37)	0.241	0.52 (0.17–1.37)	0.209	22 (11.00)	0.63 (0.37–1.06)	0.092	0.55 (0.31–0.97)	**0.043**
Dominant model			1.52 (0.68–4.05)	0.352	1.52 (0.62–4.39)	0.394		1.64 (1.02–2.74)	**0.049**	1.82 (1.09–3.17)	**0.027**
Recessive model			0.90 (0.50–1.59)	0.707	0.77 (0.40–1.44)	0.412		1.21 (0.88–1.66)	0.253	1.19 (0.84–1.69)	0.327
Additive model			1.12 (0.63–2.00)	0.707	1.31 (0.69–2.50)	0.412		0.83 (0.60–1.14)	0.253	0.84 (0.59–1.19)	0.327
*TRPV3*-rs7217270 G>A											
GG	535 (89.17)	44 (86.28)	ref		ref		166 (83.00)	ref		ref	
AG	62 (10.33)	7 (13.73)	1.37 (0.55–3.00)	0.460	1.19 (0.43–2.92)	0.716	31 (15.50)	1.61 (1.00–2.55)	**0.044**	1.58 (0.94–2.60)	0.077
AA	3 (0.50)	0 (0.00)	—	—	—	—	3 (1.50)	3.22 (0.59–17.56)	0.154	2.66 (0.46–15.78)	0.258
Dominant model			1.31 (0.52–2.86)	0.528	1.16 (0.42–2.82)	0.761		1.69 (1.07–2.63)	**0.023**	1.61 (0.98–2.60)	0.056
Recessive model			1.38 (0.55–3.02)	0.452	1.20 (0.43–2.93)	0.712		1.59 (0.99–2.52)	0.050	1.56 (0.94–2.58)	0.083
Additive model			0.72 (0.33–1.82)	0.452	0.84 (0.34–2.32)	0.712		0.63 (0.40–1.01)	0.050	0.64 (0.39–1.07)	0.083
*TRPM8*-rs17862920 C>T											
CC	321 (53.50)	31 (60.78)	ref		ref		126 (63.00)	ref		ref	
CT	237 (39.50)	16 (31.37)	0.70 (0.37–1.29)	0.263	0.70 (0.34–1.39)	0.316	66 (33.00)	0.71 (0.50–1.00)	**0.049**	0.77 (0.53–1.12)	0.169
TT	42 (7.00)	4 (7.84)	0.99 (0.28–2.65)	0.980	1.00 (0.25–3.15)	0.996	8 (4.00)	0.49 (0.21–1.01)	0.071	0.57 (0.23–1.25)	0.183
Dominant model			0.88 (0.34–3.03)	0.822	0.88 (0.29–3.43)	0.834		1.81 (0.88–4.21)	0.134	1.59 (0.73–3.89)	0.268
Recessive model			0.70 (0.37–1.27)	0.255	0.70 (0.34–1.37)	0.307		0.75 (0.54–1.05)	0.101	0.81 (0.56–1.16)	0.258
Additive model			1.43 (0.79–2.70)	0.255	1.43 (0.73–2.93)	0.307		1.33 (0.95–1.86)	0.101	1.24 (0.86–1.788)	0.258
*TRPM8*-rs7577262 G>A											
GG	220 (36.67)	27 (52.94)	ref		ref		89 (44.50)	ref		ref	
GA	294 (49.00)	17 (33.33)	0.47 (0.25–0.88)	**0.019**	0.55 (0.27–1.07)	0.081	88 (44.00)	0.74 (0.53–1.04)	0.085	0.81 (0.56–1.17)	0.257
AA	86 (14.33)	7 (13.73)	0.66 (0.26–1.50)	0.354	0.54 (0.19–1.36)	0.216	23 (11.50)	0.66 (0.39–1.10)	0.120	0.72 (0.41–1.24)	0.249
Dominant model			1.05 (0.49–2.62)	0.905	1.43 (0.59–4.02)	0.463		1.29 (0.80–2.15)	0.313	1.24 (0.75–2.13)	0.421
Recessive model			0.52 (0.28–0.94)	**0.034**	0.63 (0.32–1.19)	0.160		0.82 (0.59–1.13)	0.221	0.87 (0.62–1.24)	0.446
Additive model			1.92 (1.07–3.59)	**0.034**	1.60 (0.84–3.12)	0.160		1.22 (0.89–1.69)	0.221	1.14 (0.81–1.62)	0.446

Bold type indicates *p* < 0.05. Abbreviations: MA, migraine with aura; MO, Migraine without aura. OR_adj_ (95% CI) adjusted factors: age, sex, BMI, marital status, nationality, educational level, smoking, alcohol, exercise, history of hypertension, history of diabetes, family history of diabetes, family history of migraine, SAS grade, PHQ9 grade, PSQI grade.

*SLC17A8*, solute carrier family 17 member 8; *SHANK1*, SH3 and multiple ankyrin repeat domains 1; *TRPV1*, transient receptor potential cation channel subfamily V member 1; *TRPV3*, transient receptor potential cation channel subfamily V member 3; *TRPM8*, transient receptor potential cation channel subfamily M member 8.

Consistent with the above results, the results of matched subjects showed that three SNPs (*SLC17A8* rs11110359, *SLC17A8* rs11568537, and *TRPV3* rs7217270) were associated with MO, and *TRPM8* rs7577262 was associated with MA. Differently, *TRPV1* rs8065080 was associated with MA rather than MO. And *GRIK2* rs2227283 was associated with MA rather than migraine (Supplementary Table S9).

### 3.4 Association between gene polymorphisms and anxiety and depression

The relationship between the gene polymorphisms of TRP channels and glutamatergic synapse genes and migraine comorbidities risk was demonstrated by stratification analysis, as shown in Table 5; Supplementary Tables S4–S6. In migraine patients, the *TRPM8* rs7577262 was associated with decreased anxiety risk in a dominant model (OR_adj_ = 0.27, 95% CI = 0.10–0.76, *p* = 0.011). The genotype GA of *TRPV1* rs222741 was associated with increased anxiety risk as compared to the AA genotype (OR_adj_ = 2.72, 95% CI = 1.26–5.99, *p* = 0.011). *TRPV1* rs222741 was associated with both anxiety risk and depression risk in the recessive model (OR_adj_ = 2.64, 95% CI = 1.24–5.73, *p* = 0.012; OR_adj_ = 1.97, 95% CI = 1.02–3.85, *p* = 0.046, respectively) and additive model (OR_adj_ = 0.38, 95% CI = 0.17–0.81, *p* = 0.012; OR_adj_ = 0.51, 95% CI = 0.26–0.98, *p* = 0.046, respectively). *SLC17A8* rs11110359, *TRPV4* rs3742037 and *TRPM8* rs17862920 were associated with depression in dominant model (OR_adj_ = 0.42, 95% CI = 0.20–0.84, *p* = 0.016; OR_adj_ = 2.03, 95% CI = 1.06–3.96, *p* = 0.035; OR_adj_ = 0.48, 95% CI = 0.23–0.96, *p* = 0.042, respectively). *TRPM8*-rs10166942 was associated with depression risk in recessive model (OR_adj_ = 0.51, 95% CI = 0.27–0.94, *p* = 0.034) and additive model (OR_adj_ = 1.98, 95% CI = 1.06–3.77, *p* = 0.034) (Table 5). Other not significant results were shown in Supplementary Table S4. In nonmigraine subjects, only *SHANK2* rs55678639 was associated with depression (Supplementary Table S5). In anxiety or depression patients, *TRPM8* rs7577262 was still associated with migraine (Supplementary Table S6).

**TABLE 5:** Associations between *SLC17A8, TRPV1*, *TRPV4* and *TRPM8* gene polymorphisms and anxiety and depression in migraine patients.

SNPs/genotypes/genetic models	Anxiety No. (%)		OR_adj_ (95% CI)	*p*	Depression No. (%)		OR_adj_ (95% CI)	*p*
	No anxiety	Anxiety			No depression	Depression		
*SLC17A8*-rs11110359 G>A								
GG	147 (72.77)	36 (73.47)	ref		95 (68.35)	88 (78.57)	ref	
GA	51 (25.25)	12 (24.49)	0.66 (0.27–1.51)	0.338	41 (29.50)	22 (19.64)	0.43 (0.20–0.88)	**0.024**
AA	4 (1.98)	1 (2.04)	3.26 (0.15–31.27)	0.341	3 (2.16)	2 (1.79)	0.29 (0.02–2.91)	0.300
Dominant model			0.74 (0.31–1.64)	0.464			0.42 (0.20–0.84)	**0.016**
Recessive model			0.65 (0.27–1.48)	0.319			0.44 (0.21–0.90)	**0.028**
Additive model			1.54 (0.68–3.77)	0.319			2.28 (1.11–4.82)	**0.028**
*TRPV1*-rs222741 A>G								
AA	137 (67.82)	25 (51.02)	ref		98 (70.50)	64 (57.14)	ref	
GA	59 (29.21)	23 (46.94)	2.72 (1.26–5.99)	**0.011**	37 (26.62)	45 (40.18)	1.98 (1.02–3.89)	**0.046**
GG	6 (2.97)	1 (2.04)	1.67 (0.08–11.99)	0.659	4 (2.88)	3 (2.68)	1.12 (0.15–7.19)	0.909
Dominant model			0.93 (0.28–3.61)	0.906			0.70 (0.24–2.04)	0.503
Recessive model			2.64 (1.24–5.73)	**0.012**			1.97 (1.02–3.85)	**0.046**
Additive model			0.38 (0.17–0.81)	**0.012**			0.51 (0.26–0.98)	**0.046**
*TRPV4*-rs3742037 G>A								
GG	136 (67.33)	32 (65.31)	ref		99 (71.22)	69 (61.61)	ref	
GA	59 (29.21)	14 (28.57)	1.23 (0.54–2.78)	0.615	36 (25.90)	37 (33.04)	2.03 (1.02–4.09)	**0.045**
AA	7 (3.47)	3 (6.12)	1.15 (0.18–5.92)	0.877	4 (2.88)	6 (5.36)	2.06 (0.43–11.10)	0.373
Dominant model			1.22 (0.56–2.63)	0.614			2.03 (1.06–3.96)	**0.035**
Recessive model			1.22 (0.54–2.74)	0.626			1.95 (0.99–3.92)	0.055
Additive model			0.82 (0.37–1.87)	0.626			0.51 (0.26–1.01)	0.055
*TRPM8*-rs17862920 C>T								
CC	130 (64.36)	27 (55.10)	ref		84 (60.43)	73 (65.18)	ref	
CT	64 (31.68)	18 (36.74)	2.00 (0.87–4.65)	0.102	50 (35.97)	32 (28.57)	0.84 (0.42–1.69)	0.628
TT	8 (3.96)	4 (8.16)	4.72 (0.89–24.14)	0.060	5 (3.60)	7 (6.25)	3.98 (0.92–18.96)	0.070
Dominant model			0.88 (0.37–2.02)	0.769			0.48 (0.23–0.96)	**0.042**
Recessive model			1.71 (0.76–3.84)	0.193			0.75 (0.38–1.47)	0.403
Additive model			0.59 (0.26–1.32)	0.193			1.34 (0.68–2.66)	0.403
*TRPM8*-rs10166942 C>T								
CC	74 (36.63)	22 (44.90)	ref		48 (34.53)	48 (42.86)	ref	
TC	98 (48.52)	20 (40.82)	0.46 (0.20–1.04)	0.065	73 (52.52)	45 (40.18)	0.50 (0.25–0.99)	**0.048**
TT	30 (14.85)	7 (14.29)	0.99 (0.31–2.92)	0.989	18 (12.95)	19 (16.96)	0.95 (0.37–2.42)	0.909
Dominant model			0.56 (0.26–1.19)	0.131			0.59 (0.30–1.12)	0.106
Recessive model			0.46 (0.21–0.99)	0.052			0.51 (0.27–0.94)	**0.034**
Additive model			2.17 (1.01–4.87)	0.052			1.98 (1.06–3.77)	**0.034**
*TRPM8*-rs7577262 G>A								
GG	95 (47.03)	21 (42.86)	ref		58 (41.73)	58 (51.79)	ref	
GA	88 (43.56)	17 (34.69)	0.85 (0.37–1.95)	0.705	67 (48.20)	38 (33.93)	0.72 (0.36–1.40)	0.329
AA	19 (9.41)	11 (22.45)	3.41 (1.16–10.01)	**0.025**	14 (10.07)	16 (14.29)	1.47 (0.54–4.10)	0.450
Dominant model			0.27 (0.10–0.76)	**0.011**			0.58 (0.22–1.50)	0.266
Recessive model			0.64 (0.29–1.36)	0.251			0.66 (0.35–1.25)	0.203
Additive model			1.57 (0.73–3.49)	0.251			1.52 (0.80–2.89)	0.203

Bold type indicates *p* < 0.05. OR_adj_ (95% CI) adjusted factors: age, sex, BMI, marital status, nationality, educational level, smoking, alcohol, exercise, history of hypertension, history of diabetes, family history of diabetes, family history of migraine, PSQI grade.

*SLC17A8*, solute carrier family 17 member 8; *TRPV1*, transient receptor potential cation channel subfamily V member 1; *TRPV4*, transient receptor potential cation channel subfamily V member 4; *TRPM8*, transient receptor potential cation channel subfamily M member 8.

### 3.5 GRS and migraine and the comorbidities of anxiety and depression

The GRS was significantly higher in the migraine group compared with the control group (mean GRS: 11.47 ± 2.45 vs. 10.91 ± 2.42, *p* = 0.002) and there was a positive trend (*p* trend = 0.002). Compared to the GRS scores of the Q1 (0–9) group, individuals with GRS scores of Q4 (14–17) had a higher risk of migraine (OR_adj_ = 2.31, 95% CI = 1.39–3.86, *p* = 0.001) but not significant in GRS scores of Q2 (10–11) groups (OR_adj_ = 1.41, 95% CI = 0.90–2.21, *p* = 0.136) or Q3 (12–13) groups (OR_adj_ = 1.41, 95% CI = 0.89–2.24, *p* = 0.149) (Table 6). However, GRS scores of Q3 and Q4 groups had a lower risk of the comorbidity of anxiety than GRS scores of Q1 group (OR_adj_ = 0.31, 95% CI = 0.11–0.89, *p* = 0.032; OR_adj_ = 0.28, 95% CI = 0.08–0.89, *p* = 0.034, respectively) and the *p* trend was 0.020 (Table 7). A significant GRS score risk trend for comorbid depression was found (*p* trend = 0.025) though not significant in three GRS groups (*p* = 0.939, *p* = 0.137, *p* = 0.068, respectively) (Table 7).

**TABLE 6:** The relationship between the GRS and migraine susceptibility.

GRS	Total (N = 851)		T or OR_adj_ (95% CI)	*p*	Anxiety or depression (N = 281)		T or OR_adj_ (95% CI)^*^	*p*
	Control (N = 600)	Migraine (N = 251)			Control (N = 158)	Migraine (N = 123)		
Mean ± SD	10.91 ± 2.42	11.47 ± 2.45	−3.086	0.002	11.04 ± 2.31	11.22 ± 2.41	−0.616	0.539
Groups, N (%)								
Q1 (4–9)	164 (27.33)	51 (20.32)	ref		36 (22.79)	30 (24.39)	ref	
Q2 (10–11)	189 (31.50)	78 (31.08)	1.41 (0.90–2.21)	0.136	55 (34.81)	42 (34.15)	1.21 (0.60–2.45)	0.602
Q3 (12–13)	167 (27.83)	67 (26.69)	1.41 (0.89–2.24)	0.149	45 (28.48)	27 (21.95)	0.85 (0.40–1.81)	0.676
Q4 (14–17)	80 (13.33)	55 (21.91)	2.31 (1.39–3.86)	**0.001**	22 (13.92)	24 (19.51)	1.39 (0.61–3.23)	0.436
*p* for trend				**0.003**				0.743

GRS: genetic risk scores. Total of 11 SNPs (rs11110359 and rs11568537 of *SLC17A8*; *GRIK2* rs2227283; *SHANK1* rs3745521; rs8065080 and rs222741 of *TRPV1*; *TRPV3* rs7217270; *TRPV4* rs3742037; rs17862920, rs10166942 and rs7577262 of *TRPM8*) were included in the calculation of GRS. OR_adj_ (95% CI) adjusted factors: age, sex, BMI, marital status, nationality, educational level, smoking, alcohol, exercise, history of hypertension, history of diabetes, family history of diabetes, family history of migraine, SAS grade, PHQ9 grade, PSQI grade.

OR_adj_(95% CI)* adjusted factors did not include SAS grade, PHQ9 grade.

**TABLE 7:** The relationship between the GRS and migraine comorbidities of anxiety and depression susceptibility.

GRS	Migraine (N = 251)		T or OR_adj_ (95% CI)	*p*	Migraine (N = 251)		T or OR_adj_ (95% CI)	*p*
	No(N = 202)	Anxiety (N = 49)			No(N = 139)	Depression (N = 112)		
Mean ± SD	11.59 ± 2.45	11.00 ± 2.42	1.528	0.131	11.68 ± 2.47	11.22 ± 2.40	1.466	0.144
Groups, N (%)								
Q1 (4–9)	37 (18.32)	14 (28.57)	ref		23 (16.55)	28 (25.00)	ref	
Q2 (10–11)	62 (30.69)	16 (32.65)	0.48 (0.17–1.30)	0.149	40 (28.78)	38 (33.93)	0.97 (0.40–2.36)	0.939
Q3 (12–13)	56 (27.72)	11 (22.45)	0.31 (0.11–0.89)	**0.032**	44 (31.66)	23 (20.54)	0.14 (0.19–1.25)	0.137
Q4 (14–17)	47 (23.27)	8 (16.33)	0.28 (0.08–0.88)	**0.034**	32 (23.02)	23 (20.54)	0.07 (0.15–1.06)	0.068
*p* for trend				**0.020**				**0.025**

GRS: genetic risk scores. Total of 11 SNPs (rs11110359 and rs11568537 of *SLC17A8*; *GRIK2* rs2227283; *SHANK1* rs3745521; rs8065080 and rs222741 of *TRPV1*; *TRPV3* rs7217270; *TRPV4* rs3742037; rs17862920, rs10166942 and rs7577262 of *TRPM8*) were included in the calculation of GRS. OR_adj_ (95% CI) adjusted factors: age, sex, BMI, marital status, nationality, educational level, smoking, alcohol, exercise, history of hypertension, history of diabetes, family history of diabetes, family history of migraine, PSQI grade.

### 3.6 Gene-gene interaction, gene-comorbidity interaction and migraine

**FIGURE 1:**
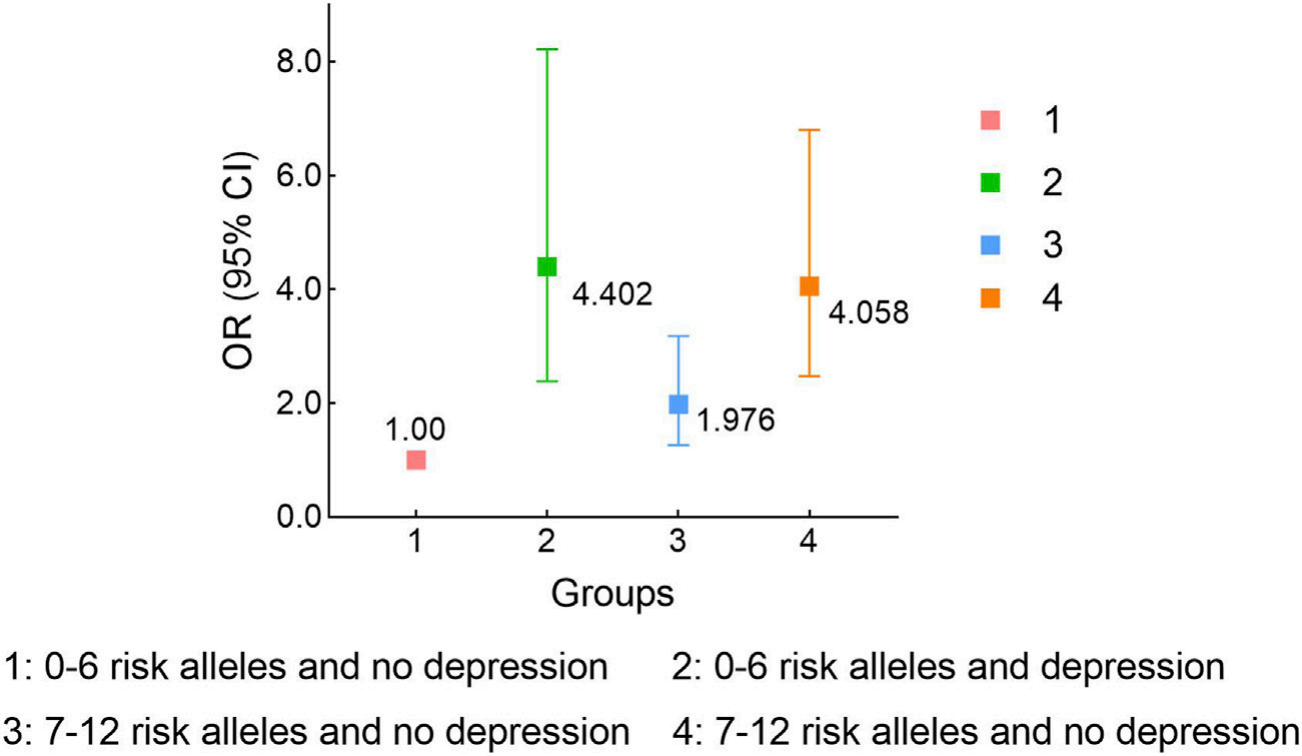
Stratified analysis for rs11568537, rs17862920, rs2227283, rs10166942, rs3745521, rs8065080 and depression interaction on migraine risk using logistic regression. Adjusted for age and sex.

GMDR model was used to evaluate the effect of SNP-SNP and gene-environment interaction on migraine risk. As shown in Table 8, we found a three-locus SNP-SNP interaction model (rs11568537× rs17862920× rs2227283) contributed to the best model with the most excellent cross-validation consistency of 9/10 (*p* = 0.0010) (Supplementary Figure S1A). For gene-depression interaction, an important four-locus model (rs10166942× rs3745521× rs8065080× Depression) contributed to the best model (*p* = 0.0010) (Supplementary Figure S1B). We further assessed the combined effect of the six risk SNPs and depression on migraine risk. Compared with subjects with no depression and zero to six risk alleles, migraine patients with depression and seven to twelve risk alleles had the highest migraine risk (OR_adj_ = 4.06, 95% CI = 2.48–6.81, *p* < 0.001) (Figure 1).

**TABLE 8:** GMDR analysis for the best SNP-SNP and gene-depression interaction models.

Locus No.	Best combination	Training	Testing	CV	Sign test (*p*)
		Accuracy	Accuracy	Consistency	
SNP-SNP interactions					
1	rs7577262	0.5472	0.5034	6/10	4 (0.8281)
2	rs17862920 rs2227283	0.5704	0.5313	8/10	8 (0.0547)
3	rs11568537 rs17862920 rs2227283	0.6034	0.5630	9/10	10 (**0.0010**) **^a^**
Gene-depression interactions					
1	Depression	0.5988	0.5932	10/10	9 (**0.0107**) **^a^**
2	rs11110359 Depression	0.6123	0.5581	3/10	8 (0.0547)
3	rs11110359 rs55678639 Depression	0.6365	0.5917	5/10	9 (**0.0107**) **^a^**
4	rs10166942 rs3745521 rs8065080 Depression	0.6783	0.5963	9/10	10 (**0.0010**) **^a^**

Bold type indicates *p* < 0.05. Abbreviations: CV, Cross validation; GMDR, Generalized multifactor dimension reduction.

^a^ *p* adjusted factors: age, sex.

### 3.7 Functional predictions

We identified seven significant sQTL signals for rs8065080 on *TRPV1* (Table 9). The C allele of rs8065080 was related to the increased level of alternative splicing of the pre-mRNA for gene *TRPV1* from multiple types of human tissues. In addition, significant eQTL signals were identified for SNPs rs2227283, rs7577262, rs8065080 and rs7217270 (Supplementary Tables S10–S13). The threshold of *p* values was 0.005 (0.05/10) or 0.001 (0.05/46 or 0.05/49). Four significant signals (4/49) were identified for SNP rs8065080 and gene expression levels of *TRPV1* (Supplementary Table S1). The T allele of rs8065080 was significantly associated with an increased gene expression level of *TRPV1* in the skin-sun exposed (lower leg) (Supplementary Figure S2A, *p* = 2.30 × 10^−5^) and in the skin-not Sun exposed (suprapubic) (SSupplementary Figure S2B, *p* = 5.20 × 10^−5^). However, no statistically significant was found between *TRPV1* gene expression and neural or brain tissues.

**TABLE 9:** Significant sQTL signals of SNP rs8065080 on *TRPV1*.

Gene	Ref_Alt alleles	SNP	*p*-value	NES	Tissue
*TRPV1*	T_C	rs8065080	1.50E-34	0.71	Nerve—Tibial
*TRPV1*	T_C	rs8065080	2.30E-12	0.54	Testis
*TRPV1*	T_C	rs8065080	3.00E-09	0.67	Brain—Cerebellar Hemisphere
*TRPV1*	T_C	rs8065080	1.80E-08	0.57	Brain—Cerebellum
*TRPV1*	T_C	rs8065080	3.40E-08	0.6	Ovary
*TRPV1*	T_C	rs8065080	1.50E-07	−0.32	Adipose—Subcutaneous
*TRPV1*	T_C	rs8065080	1.20E-06	−0.31	Adipose—Visceral (Omentum)

Ref_Alt Alleles: reference and alternative alleles.

*TRPV1*, transient receptor potential cation channel subfamily V member 1.

In addition, we found that *SLC17A8* rs11110359 and *TRPV1* rs8065080 are located at exonic splicing enhancer (ESE) or exonic splicing silencer (ESS) -binding sites (Supplementary Table S1). We found several microRNA binding sites For *SLC17A8* rs11568537 and *SHANK2* rs55678639 (Supplementary Table S5).

### 3.8 Power analysis

We calculated the power of the study for 13 SNPs using odds ratios (OR) of 1.8, 2.0, and 3.0 to assess whether the sample size of this study was sufficient to be biologically meaningful. Except for SNP rs7217270, the power analysis indicated that our sample size achieved sufficient power (>0.831) to conclude with OR 1.8 or higher (Table 10). As for subgroup analysis, except for SNP rs7217270 and SNP rs11110359, the power was higher than 80% in groups of MO vs. Control and not approaching 80% in groups of MA vs. Control with OR 1.8 or higher (Table 10).

**TABLE 10:** Power of the study with different odds ratios (OR) in an allelic model.

Gene	SNP	MAF	Migraine vs. Control			MA vs. Control			MO vs. Control		
			OR = 1.8	OR = 2	OR = 3	OR = 1.8	OR = 2	OR = 3	OR = 1.8	OR = 2	OR = 3
*SLC17A8*	rs11110359	0.138	0.853	0.947	1	0.477	0.523	0.894	0.797	0.912	1
*SLC17A8*	rs11568537	0.180	0.908	0.974	1	0.547	0.578	0.929	0.861	0.951	1
*GRM4*	rs2229900	0.435	0.974	0.996	1	0.699	0.658	0.963	0.949	0.989	1
*GRIK2*	rs2227283	0.347	0.971	0.995	1	0.684	0.665	0.966	0.944	0.987	1
*GRIA3*	rs3761555	0.349	0.971	0.995	1	0.685	0.665	0.966	0.944	0.987	1
*SHANK1*	rs3745521	0.456	0.974	0.996	1	0.698	0.652	0.961	0.948	0.988	1
*SHANK2*	rs3020047	0.127	0.831	0.935	1	0.455	0.506	0.880	0.774	0.896	1
*SHANK2*	rs55678639	0.203	0.927	0.982	1	0.578	0.600	0.941	0.884	0.963	1
*TRPV1*	rs8065080	0.394	0.974	0.996	1	0.696	0.665	0.967	0.948	0.989	1
*TRPV1*	rs222741	0.193	0.919	0.979	1	0.565	0.591	0.936	0.875	0.958	1
*TRPV3*	rs7217270	0.063	0.605	0.756	0.992	0.296	0.367	0.722	0.550	0.698	0.982
*TRPV4*	rs3742037	0.181	0.909	0.975	1	0.549	0.579	0.930	0.862	0.952	1
*TRPM8*	rs17862920	0.250	0.951	0.990	1	0.627	0.634	0.956	0.916	0.977	1
near *TRPM8*	rs10166942	0.355	0.971	0.995	1	0.687	0.666	0.966	0.945	0.988	1
*TRPM8*	rs7577262	0.373	0.973	0.996	1	0.692	0.666	0.966	0.947	0.988	1

MAF: minor allele frequency.

## 4 Discussion

### 4.1 Mainly findings

In this case control study, the *GRIK2* rs2227283 was the first time studied and found to be associated with migraine risk in the edge of significance. More importantly, we first found that *TRPV1* rs222741 was associated with migraine comorbidities both anxiety and depression. *TRPM8* rs7577262 was associated with migraine comorbidity anxiety. *TRPV4* rs3742037, *TRPM8* rs17862920, *TRPM8* rs10466942 and *SLC17A8* rs11110359 were associated with migraine comorbidity depression risk. In anxiety or depression patients, rs7577262 was still related to the susceptibility of migraine. However, in controls, only *SHANK2* rs55678639 was associated with depression. These results further indicate that migraine and its comorbidities of anxiety and depression may share common genetic traits.

Further, we found one novel *SHANK1* rs3745521 was associated with migraine with aura risk, and two novel SNPs (rs11110359 and rs11568537 of *SLC17A8* gene) were associated with the susceptibility of migraine without aura risk. The present study showed that *TRPV1* rs8065080, *TRPV3* rs7217270, and *TRPM8* rs17862920 were significantly associated with migraine or MO risk. *TRPM8* rs7577262 was associated with migraine or MA risk. These findings confirm the results of previous studies (Carreño et al., 2012; Chasman et al., 2014; Fu et al., 2019; Gavva et al., 2019). However, the associations between MA and MO may not be trustworthy enough for the sample size in the subgroups was not so large. There are no common SNPs associated with both MA and MO in these two pathways, suggesting that different pathways may be involved in the MA/MO susceptibility.

### 4.2 Previously studied SNPs

*TRPV1* and *TRPV4* are abundantly expressed in primary sensory neurons and play a primary role in migraine pain (Iannone et al., 2022). *TRPM8* encodes for a receptor-activated non-selective cation channel activated by cold environmental temperatures and is related to pain sensor channels (Dussor and Cao, 2016). As the main members of the TRP family, the polymorphisms of *TRPV1*, *TRPV4* and *TRPM8* genes may play an important role in the occurrence and development of migraine. rs222741 (*TRPV1*) and rs7577262 (*TRPM8*) were associated with migraine overall (Carreño et al., 2012; Chasman et al., 2014). We also found that *TRPM8* rs7577262 was associated with migraine risk. However, rs7577262 was not a risk factor for migraines in a small sample of the She ethnic population in China (Fu et al., 2019). Anxiety and depression are the most frequent psychiatric comorbidities in migraine (Bergman-Bock, 2018). We found that the TRP gene polymorphisms in migraine comorbidity of anxiety and depression were different except for *TRPV1* rs222741. It may be that the mechanisms underlying these two comorbidities are not quite identical. One study suggested that similar clinical phenotypes of anxiety and depression rely on prefrontal alterations, whereas frontotemporal and parietal abnormalities may represent unique features of the two (Maggioni et al., 2019).

### 4.3 Novel SNPs

*GRIK2* rs2227283 was associated with migraine risk in unmatched data and associated with MA risk in matched data. The *GRIK2* gene is located in the 6q16.3 region and encodes for kainate receptors (KARs) class ionotropic glutamate receptor 6 (GluR6). *GRIK2* contributes to inhibitory transmission, regulates excitatory responses and glucose homeostasis, and plays an important role in synaptic physiology (Barbon et al., 2001; Abarkan et al., 2019). Previous studies showed that the *GRIK2* gene was associated with somatic anxiety, recurrent epileptic seizures, autism, and cognitive abilities (Myung et al., 2012; Yuan et al., 2015; Chandra et al., 2019; Henley et al., 2021). A recent study provided further evidence that the rs2227283 polymorphism of the *GRIK2* gene was significantly related to aggressive behaviors in bipolar manic patients (Ma et al., 2019). Therefore, we hypothesize that polymorphisms in this gene contribute to migraine onset by affecting neurodevelopment.

This study showed that the polymorphisms of *SLC17A8* may increase MO risk. *SLC17A8* encodes the vesicular glutamate transporter 3 (VGLUT3), expressed in discrete populations of glutamatergic, cholinergic, serotonergic, and even GABAergic neurons (Favier et al., 2021). Genetic deletion of VGLUT3 triggers deficits in acute and persistent mechanical pain and inflammatory pain (Seal et al., 2009; Draxler et al., 2014). Chronic pain decreases VGLUT3 levels (Tukey et al., 2013). Moreover, Brain-derived neurotrophic factor (*BDNF*) might regulate neuropathic pain through the upregulation of VGLUT3 by activation of the phospholipase C-gamma (PLC-γ) signaling pathway (Liu et al., 2014). VGLUT-3 expression was upregulated in the conditions of diabetes complicated by depression (Liu et al., 2021). Interestingly, we found that *SLC17A8* rs11110359 polymorphisms were associated with migraine combined depression. The metabotropic glutamate receptors (mGluRs) involved in the regulation of synaptic transmission and neuronal excitability throughout the central nervous system and the targeted drug has therapeutic effect on depression (Niswender and Conn, 2010). Our findings may provide genetic evidence for this mechanism.

Our findings suggested that polymorphisms of *SHANK1* (rs3745521) might be significantly correlated with MA susceptibility. SH3 and multiple ankyrin repeat domains proteins *SHANK*1, *SHANK2*, and *SHANK3* encode a family of postsynaptic scaffolding proteins present at glutamatergic synapses and play a crucial role in synaptogenesis (Liu et al., 2022). The expression and loss of allodynia and hyperalgesia are closely associated with changes in Homer1b/c and Shank1a levels in the spinal dorsal horn (Miletic et al., 2005). Moreover, *SHANK2* knock-out mice showed reduced tactile perception and analgesia to chronic pain in autism spectrum disorder (Ko et al., 2016). The *SHANK* genes have been extensively studied in neuropsychiatric disorders such as autism and schizophrenia, but the mechanism of the role of *SHANK* in migraine is unclear (Schmeisser, 2015; Wan et al., 2022).

### 4.4 GRS and functional prediction

Moreover, our GRS analysis also revealed that the higher GRS scores increased migraine risk and decreased comorbidity anxiety risk. A significant GRS score risk trend for comorbid depression was found though nonsignificant between GRS groups, which was due to the small sample size of our cases. Furthermore, we identified one significant SNP-SNP interaction model (rs11568537× rs17862920× rs2227283) and a significant gene-depression interaction model (rs10166942× rs3745521× rs8065080× Depression) which contributed to the best model. We found that more risk loci and depression increased migraine risk, suggesting that genetic factors and migraine comorbidity depression combine to increase migraine risk. In addition, we investigated the potential functional consequences of the SNPs *GRIK2* rs2227283, *TRPV1* rs8065080, *TRPV3* rs7217270 and *TRPM8* rs7577262 through eQTL and sQTL data obtained from the GTEx database. However, these data should be interpreted with caution because samples collected by the GTEx database are from healthy individuals. We found that *SLC17A8* rs11110359 and *TRPV1* rs8065080 might disrupt mRNA splicing and severely affect protein function. *SLC17A8* rs11568537 and *SHANK2* rs55678639 might affect miRNA binding site activity.

### 4.5 Limitations

Although none of the SNPs are significant after the Bonferroni correction, the positive SNP results are still meaningful in migraine genetic studies. Some limitations of the present study should be considered. First, the sample size in this study was relatively small, especially in the subgroup analysis. Second, the migraine comorbidities in this study were not diagnosed by a physician and were only assessed by a simple scale. Finally, the biological mechanism of these gene polymorphisms associated with migraine patients remains unclear, although this study found that some SNPs are associated with migraine. Therefore, further studies are necessary to determine better the biological mechanism of the polymorphisms of TRP channels genes and glutamatergic synapse genes associated with migraine.

## 5 Conclusion

In summary, this study suggested that the polymorphisms of TRP channels and glutamatergic synapse genes may increase the risk of migraine and the risk of the comorbidities of anxiety and depression in China. Higher GRS scores may increase migraine risk and decrease comorbidity anxiety risk. Further genetic studies with large samples and mechanistic studies are needed.

## Data Availability

The original contributions presented in the study are included in the article/Supplementary Material, further inquiries can be directed to the corresponding authors.
