# Supplementary material for: Association between TRP channels and glutamatergic synapse gene polymorphisms and migraine and the comorbidities anxiety and depression in a Chinese population
Source: Front Genet. 2023 May 26;14:1158028. doi: 10.3389/fgene.2023.1158028 (PMC10250607; doi:10.3389/fgene.2023.1158028)
Supplement: Supplementary file 1 [file Table1.DOCX]

Supplementary Material

# Supplementary Figures and Tables

## Supplementary Figures


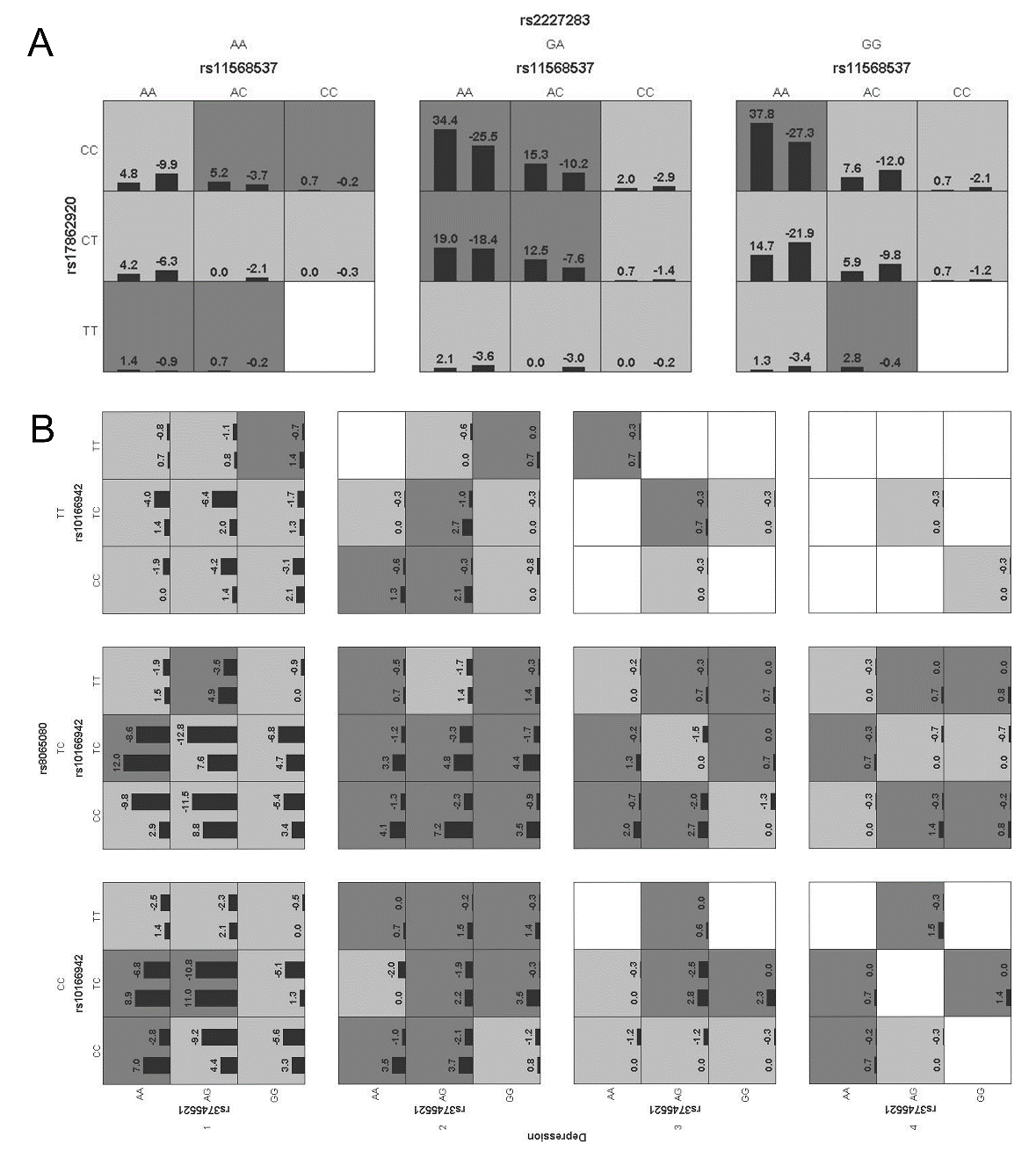


**Figure S1.** The best SNP-SNP and gene-depression interaction models. (A) Best three-way gene-gene interaction model in unmatched subjects. The best model is composed of rs11568537, rs17862920 and rs2227283. (B) The best gene-depression interaction model. The model is composed of rs10166942, rs3745521, rs8065080 and depression. The depression of 1-4 indicated no, mild, moderate, moderate to severe depression. The adjusted covariates included age and sex. In each box, the left bar represents a positive score, and the right bar a negative score. High- risk boxes are indicated by dark shading, low- risk boxes by light shading, and empty cells by no shading.


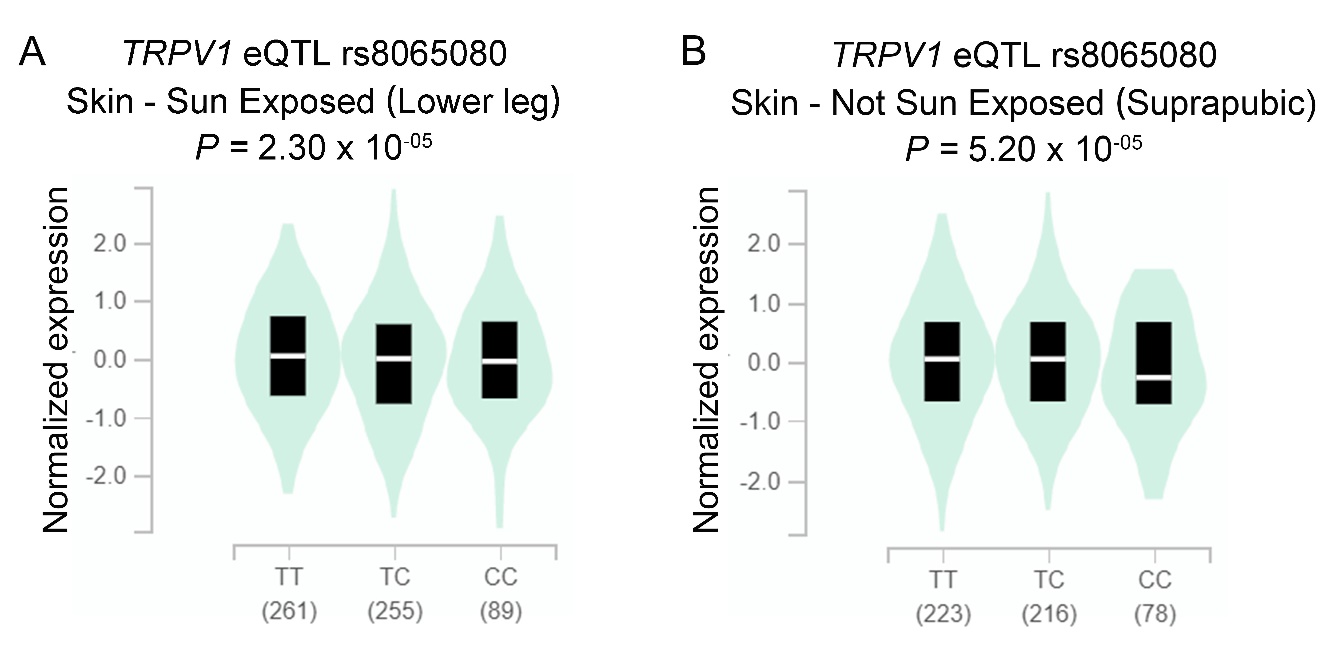


**Figure S2.** Violin plots for genotypes of rs8065080 on gene expression level of *TRPV1*. (A) and (B), Violin plot for genotypes of SNP rs8065080 on *TRPV1* gene expression levels in skin sun exposed and skin not sun exposed.

## Supplementary Tables

**Table S1.** Repeat consistency analysis on the target 15 SNPs.

| No. | SNP | Gene | Repeatability | Statistic | *P* |
| --- | --- | --- | --- | --- | --- |
| 1 | rs11110359 | SLC17A8 | 96.48% | 8.132 | <0.001 |
| 2 | rs11568537 | SLC17A8 | 95.86% | 7.764 | <0.001 |
| 3 | rs2229900 | GRM4 | 97.34% | 10.376 | <0.001 |
| 4 | rs2227283 | GRIK2 | 100.00% | 9.979 | <0.001 |
| 5 | rs3761555 | GRIA3 | 97.23% | 10.055 | <0.001 |
| 6 | rs3745521 | SHANK1 | 100.00% | 9.950 | <0.001 |
| 7 | rs3020047 | SHANK2 | 100.00% | 8.923 | <0.001 |
| 8 | rs55678639 | SHANK2 | 96.48% | 9.180 | <0.001 |
| 9 | rs8065080 | TRPV1 | 97.05% | 9.505 | <0.001 |
| 10 | rs222741 | TRPV1 | 100.00% | 8.831 | <0.001 |
| 11 | rs7217270 | TRPV3 | 100.00% | 7.616 | <0.001 |
| 12 | rs3742037 | TRPV4 | 100.00% | 8.987 | <0.001 |
| 13 | rs17862920 | TRPM8 | 96.77% | 8.445 | <0.001 |
| 14 | rs10166942 | near TRPM8 | 100.00% | 9.949 | <0.001 |
| 15 | rs7577262 | TRPM8 | 97.12% | 9.865 | <0.001 |

7% (58/851) of samples were randomly selected and genotyped twice.

**Table S2.** Associations between the gene polymorphisms of TRP channels and Glutamatergic synapse genes and the risk of migraine in unmatched subjects (*P*>0.05).

| SNPs/genotypes/genetic models | Control No. (%) | Migraine No. (%) | OR (95% CI) | *P* |  | OR_adj_ (95% CI) | *P* |
| --- | --- | --- | --- | --- | --- | --- | --- |
| TRPM8-rs10166942 C>T |  |  |  |  |  |  |  |
| CC | 253 (42.17) | 96 (38.25) | ref |  |  | ref |  |
| TC | 281 (46.83) | 118 (47.01) | 1.11 (0.81-1.52) | 0.533 |  | 1.16 (0.82-1.65) | 0.394 |
| TT | 66 (11.00) | 37 (14.74) | 1.48 (0.92-2.35) | 0.101 |  | 1.41 (0.84-2.34) | 0.187 |
| Dominant model |  |  | 1.18 (0.87-1.60) | 0.289 |  | 1.21 (0.87-1.69) | 0.250 |
| Recessive model |  |  | 1.01 (0.75-1.35) | 0.962 |  | 1.07 (0.78-1.48) | 0.677 |
| Additive model |  |  | 0.99 (0.74-1.34) | 0.962 |  | 0.93 (0.68-1.29) | 0.677 |
| *SLC17A8*-rs11110359 G>A |  |  |  |  |  |  |  |
| GG | 462 (77.00) | 183 (72.91) | ref |  |  | ref |  |
| GA | 122 (20.33) | 63 (25.10) | 1.30 (0.92-1.84) | 0.136 |  | 1.35 (0.91-1.97) | 0.132 |
| AA | 16 (2.67) | 5 (1.99) | 0.79 (0.26-2.05) | 0.648 |  | 0.88 (0.27-2.48) | 0.824 |
| Dominant model |  |  | 1.24 (0.89-1.74) | 0.204 |  | 1.29 (0.89-1.87) | 0.177 |
| Recessive model |  |  | 1.31 (0.92-1.85) | 0.125 |  | 1.35 (0.92-1.98) | 0.126 |
| Additive model |  |  | 0.76 (0.54-1.08) | 0.125 |  | 0.74 (0.51-1.09) | 0.126 |
| *SLC17A8*-rs11568537 A>C |  |  |  |  |  |  |  |
| AA | 404 (67.33) | 173 (68.92) | ref |  |  | ref |  |
| AC | 167 (27.83) | 71 (28.29) | 0.99 (0.71-1.38) | 0.966 |  | 1.01 (0.70-1.44) | 0.974 |
| CC | 29 (4.83) | 7 (2.79) | 0.56 (0.22-1.24) | 0.183 |  | 0.49 (0.18-1.17) | 0.128 |
| Dominant model |  |  | 1.77 (0.81-4.44) | 0.182 |  | 2.04 (0.87-5.46) | 0.124 |
| Recessive model |  |  | 1.02 (0.73-1.42) | 0.893 |  | 1.04 (0.73-1.49) | 0.817 |
| Additive model |  |  | 0.98 (0.71-1.36) | 0.893 |  | 0.96 (0.67-1.37) | 0.817 |
| *TRPV1*-rs222741 A>G |  |  |  |  |  |  |  |
| AA | 387 (64.50) | 162 (64.54) | ref |  |  | ref |  |
| GA | 194 (32.33) | 82 (32.67) | 1.01 (0.73-1.38) | 0.952 |  | 0.90 (0.64-1.28) | 0.563 |
| GG | 19 (3.17) | 7 (2.79) | 0.88 (0.34-2.05) | 0.778 |  | 0.71 (0.26-1.76) | 0.473 |
| Dominant model |  |  | 1.14 (0.49-2.95) | 0.770 |  | 1.361 (0.56-3.72) | 0.516 |
| Recessive model |  |  | 1.02 (0.74-1.39) | 0.924 |  | 0.92 (0.65-1.29) | 0.626 |
| Additive model |  |  | 0.99 (0.72-1.35) | 0.924 |  | 1.09 (0.77-1.55) | 0.626 |
| *SHANK2*-rs3020047 C>T |  |  |  |  |  |  |  |
| CC | 464 (77.33) | 182 (72.51) | ref |  |  | ref |  |
| TC | 126 (21.00) | 65 (25.90) | 1.32 (0.93-1.85) | 0.120 |  | 1.14 (0.78-1.67) | 0.495 |
| TT | 10 (1.67) | 4 (1.59) | 1.02 (0.28-3.09) | 0.974 |  | 1.01 (0.25-3.36) | 0.992 |
| Dominant model |  |  | 1.29 (0.92-1.81) | 0.134 |  | 1.13 (0.78-1.64) | 0.513 |
| Recessive model |  |  | 1.32 (0.93-1.85) | 0.119 |  | 1.14 (0.78-1.67) | 0.495 |
| Additive model |  |  | 0.76 (0.54-1.08) | 0.119 |  | 0.88 (0.60-1.29) | 0.495 |
| *TRPV4*-rs3742037 G>A |  |  |  |  |  |  |  |
| GG | 404 (67.33) | 168 (66.93) | ref |  |  | ref |  |
| GA | 175 (29.17) | 73 (29.08) | 1.00 (0.72-1.39) | 0.985 |  | 0.91 (0.64-1.30) | 0.608 |
| AA | 21 (3.50) | 10 (3.98) | 1.15 (0.51-2.43) | 0.732 |  | 1.05 (0.43-2.38) | 0.920 |
| Dominant model |  |  | 1.02 (0.74-1.39) | 0.910 |  | 0.93 (0.66-1.30) | 0.655 |
| Recessive model |  |  | 1.00 (0.72-1.37) | 0.981 |  | 0.91 (0.64-1.29) | 0.596 |
| Additive model |  |  | 1.00 (0.73-1.39) | 0.981 |  | 1.10 (0.77-1.58) | 0.596 |
| *SHANK1*-rs3745521 A>G |  |  |  |  |  |  |  |
| AA | 173 (28.83) | 80 (31.87) | ref |  |  | ref |  |
| AG | 293 (48.83) | 114 (45.42) | 0.84 (0.60-1.19) | 0.322 |  | 0.72 (0.50-1.05) | 0.091 |
| GG | 134 (22.33) | 57 (22.71) | 0.92 (0.61-1.38) | 0.688 |  | 0.79 (0.50-1.23) | 0.302 |
| Dominant model |  |  | 0.98 (0.69-1.40) | 0.905 |  | 1.04 (0.71-1.53) | 0.859 |
| Recessive model |  |  | 0.87 (0.65-1.17) | 0.363 |  | 0.80 (0.58-1.11) | 0.179 |
| Additive model |  |  | 1.15 (0.85-1.54) | 0.363 |  | 1.25 (0.91-1.72) | 0.179 |
| *SHANK2*-rs55678639 A>G |  |  |  |  |  |  |  |
| AA | 380 (63.33) | 168 (66.93) | ref |  |  | ref |  |
| AG | 189 (31.50) | 72 (28.69) | 0.86 (0.62-1.19) | 0.372 |  | 0.90 (0.63-1.29) | 0.581 |
| GG | 31 (5.17) | 11 (4.38) | 0.80 (0.38-1.59) | 0.545 |  | 0.76 (0.34-1.59) | 0.475 |
| Dominant model |  |  | 1.19 (0.61-2.51) | 0.630 |  | 1.29 (0.62-2.87) | 0.519 |
| Recessive model |  |  | 0.88 (0.63-1.21) | 0.417 |  | 0.92 (0.64-1.31) | 0.650 |
| Additive model |  |  | 1.14 (0.83-1.59) | 0.417 |  | 1.09 (0.76-1.55) | 0.650 |

OR_adj_ (95% CI) adjusted factors: age, sex, BMI, marital status, nationality, educational level, smoking, alcohol, exercise, history of hypertension, history of diabetes, family history of diabetes, family history of migraine, SAS grade, PHQ9 grade, PSQI grade.

**Table S3.** Associations between the gene polymorphisms of TRP channels and glutamatergic synapse genes and the risk of migraine by aura in unmatched subjects (*P*>0.05).

| SNPs/genotypes/  genetic models | Control  No. (%) | MA | | | | |  | MO | | | | |
| --- | --- | --- | --- | --- | --- | --- | --- | --- | --- | --- | --- | --- |
|  |  | No. (%) | OR (95% CI) | *P* | OR_adj_ (95% CI) | *P* |  | No. (%) | OR (95% CI) | *P* | OR_adj_ (95% CI) | *P* |
| *TRPM8*-rs10166942 C>T |  |  |  |  |  |  |  |  |  |  |  |  |
| CC | 253 (42.17) | 19 (37.26) | ref |  | ref |  |  | 77 (38.50) | ref |  | ref |  |
| TC | 281 (46.83) | 22 (43.14) | 1.04 (0.55-1.99) | 0.898 | 1.34 (0.66-2.76) | 0.418 |  | 96 (48.00) | 1.12 (0.80-1.59) | 0.511 | 1.12 (0.77-1.63) | 0.551 |
| TT | 66 (11.00) | 10 (19.61) | 2.02 (0.87-4.46) | 0.090 | 2.31 (0.90-5.67) | 0.071 |  | 27 (13.50) | 1.34 (0.79-2.23) | 0.261 | 1.28 (0.73-2.21) | 0.385 |
| Dominant model |  |  | 1.23 (0.69-2.25) | 0.495 | 1.55 (0.82-3.06) | 0.189 |  |  | 1.17 (0.84-1.62) | 0.362 | 1.15 (0.81-1.64) | 0.432 |
| Recessive model |  |  | 0.86 (0.48-1.53) | 0.612 | 1.07 (0.56-2.05) | 0.828 |  |  | 1.05 (0.76-1.44) | 0.775 | 1.06 (0.75-1.50) | 0.751 |
| Additive model |  |  | 1.16 (0.65-2.09) | 0.612 | 0.93 (0.49-1.78) | 0.828 |  |  | 0.95 (0.69-1.32) | 0.775 | 0.95 (0.67-1.34) | 0.751 |
| *GRIK2*-rs2227283 G>A |  |  |  |  |  |  |  |  |  |  |  |  |
| GG | 269 (44.83) | 17 (33.33) | ref |  | ref |  |  | 86 (43.00) | ref |  | ref |  |
| GA | 249 (41.50) | 27 (52.94) | 1.72 (0.92-3.28) | 0.093 | 1.64 (0.83-3.32) | 0.162 |  | 97 (48.50) | 1.22 (0.87-1.71) | 0.251 | 1.20 (0.83-1.73) | 0.340 |
| AA | 82 (13.67) | 7 (13.73) | 1.35 (0.51-3.25) | 0.519 | 1.44 (0.50-3.79) | 0.477 |  | 17 (8.50) | 0.65 (0.36-1.13) | 0.141 | 0.64 (0.33-1.16) | 0.152 |
| Dominant model |  |  | 1.00 (0.46-2.48) | 0.991 | 0.91 (0.39-2.46) | 0.845 |  |  | 1.70 (1.01-3.04) | 0.057 | 1.73 (0.98-3.21) | 0.071 |
| Recessive model |  |  | 1.59 (0.89-2.83) | 0.115 | 1.49 (0.79-2.81) | 0.215 |  |  | 1.33 (0.96-1.83) | 0.084 | 1.31 (0.92-1.86) | 0.132 |
| Additive model |  |  | 0.63 (0.35-1.12) | 0.115 | 0.67 (0.36-1.26) | 0.215 |  |  | 0.75 (0.55-1.04) | 0.084 | 0.76 (0.54-1.09) | 0.132 |
| *TRPV1*-rs222741 A>G |  |  |  |  |  |  |  |  |  |  |  |  |
| AA | 387 (64.50) | 36 (70.59) | ref |  | ref |  |  | 126 (63.00) | ref |  | ref |  |
| GA | 194 (32.33) | 14 (27.45) | 0.78 (0.40-1.44) | 0.438 | 0.66 (0.31-1.31) | 0.248 |  | 68 (34.00) | 1.08 (0.76-1.51) | 0.672 | 0.96 (0.66-1.39) | 0.818 |
| GG | 19 (3.17) | 1 (1.96) | 0.57(0.03-2.85) | 0.584 | 0.53 (0.03-2.94) | 0.550 |  | 6 (3.00) | 0.97 (0.35-2.35) | 0.949 | 0.79 (0.27-2.06) | 0.652 |
| Dominant model |  |  | 1.64 (0.33-29.68) | 0.635 | 1.66 (0.30-31.21) | 0.636 |  |  | 1.06 (0.44-2.94) | 0.907 | 1.24 (0.48-3.64) | 0.671 |
| Recessive model |  |  | 0.79 (0.41-1.47) | 0.474 | 0.68 (0.32-1.34) | 0.277 |  |  | 1.08 (0.77-1.51) | 0.664 | 0.97 (0.67-1.40) | 0.865 |
| Additive model |  |  | 1.26 (0.68-2.47) | 0.474 | 1.48 (0.75-3.12) | 0.277 |  |  | 0.93 (0.66-1.31) | 0.664 | 1.03 (0.72-1.50) | 0.865 |
| *SHANK2*-rs3020047 C>T |  |  |  |  |  |  |  |  |  |  |  |  |
| CC | 464 (77.33) | 40 (78.43) | ref |  | ref |  |  | 142 (71.00) | ref |  | ref |  |
| TC | 126 (21.00) | 11 (21.57) | 1.01 (0.48-1.97) | 0.972 | 0.77 (0.33-1.64) | 0.513 |  | 54 (27.00) | 1.40 (0.96-2.02) | 0.075 | 1.24 (0.82-1.85) | 0.305 |
| TT | 10 (1.67) | 0 (0.00) | — | — | — | — |  | 4 (2.00) | 1.31 (0.35-3.97) | 0.655 | 1.33 (0.34-4.42) | 0.659 |
| Dominant model |  |  | 0.94 (0.45-1.82) | 0.857 | 0.71 (0.31-1.51) | 0.392 |  |  | 1.39 (0.97-1.99) | 0.071 | 1.24 (0.84-1.84) | 0.278 |
| Recessive model |  |  | 1.04 (0.49-2.01) | 0.924 | 0.79 (0.34-1.69) | 0.557 |  |  | 1.39 (0.96-2.01) | 0.079 | 1.23 (0.82-1.83) | 0.318 |
| Additive model |  |  | 0.97 (0.50-2.03) | 0.924 | 1.27 (0.59-2.95) | 0.557 |  |  | 0.72 (0.50-1.04) | 0.079 | 0.81 (0.55-1.23) | 0.318 |
| *TRPV4*-rs3742037 G>A |  |  |  |  |  |  |  |  |  |  |  |  |
| GG | 404 (67.33) | 36 (70.59) | ref |  | ref |  |  | 132 (66.00) | ref |  | ref |  |
| GA | 175 (29.17) | 11 (21.57) | 0.71 (0.34-1.37) | 0.327 | 0.66 (0.30-1.38) | 0.291 |  | 62 (31.00) | 1.08 (0.76-1.53) | 0.650 | 0.99 (0.68-1.44) | 0.962 |
| AA | 21 (3.50) | 4 (7.84) | 2.14 (0.60-5.99) | 0.185 | 1.53 (0.35-5.41) | 0.534 |  | 6 (3.00) | 0.87 (0.32-2.09) | 0.777 | 0.92 (0.32-2.32) | 0.861 |
| Dominant model |  |  | 0.86 (0.45-1.58) | 0.634 | 0.77 (0.37-1.51) | 0.456 |  |  | 1.06 (0.75-1.49) | 0.728 | 0.98 (0.68-1.41) | 0.928 |
| Recessive model |  |  | 0.67 (0.32-1.29) | 0.252 | 0.64 (0.29-1.32) | 0.247 |  |  | 1.09 (0.77-1.54) | 0.623 | 1.00 (0.68-1.44) | 0.979 |
| Additive model |  |  | 1.50 (0.78-3.13) | 0.252 | 1.56 (0.76-3.46) | 0.247 |  |  | 0.92 (0.65-1.30) | 0.623 | 1.01 (0.69-1.47) | 0.979 |
| *SHANK2*-rs55678639 A>G |  |  |  |  |  |  |  |  |  |  |  |  |
| AA | 380 (63.33) | 30 (58.82) | ref |  | ref |  |  | 138 (69.00) | ref |  | ref |  |
| AG | 189 (31.50) | 18 (35.29) | 1.21 (0.65-2.20) | 0.547 | 1.11 (0.55-2.18) | 0.762 |  | 54 (27.00) | 0.79 (0.55-1.12) | 0.191 | 0.82 (0.55-1.21) | 0.327 |
| GG | 31 (5.17) | 3 (5.88) | 1.23 (0.28-3.70) | 0.748 | 1.14 (0.24-3.87) | 0.854 |  | 8 (4.00) | 0.71 (0.30-1.51) | 0.403 | 0.70 (0.28-1.57) | 0.409 |
| Dominant model |  |  | 0.87 (0.30-3.72) | 0.826 | 0.91 (0.28-4.29) | 0.895 |  |  | 1.31 (0.62-3.10) | 0.508 | 1.35 (0.61-3.34) | 0.483 |
| Recessive model |  |  | 1.19 (0.64-2.14) | 0.577 | 1.10 (0.55-2.13) | 0.783 |  |  | 0.80 (0.56-1.14) | 0.231 | 0.84 (0.57-1.23) | 0.382 |
| Additive model |  |  | 0.84 (0.47-1.56) | 0.577 | 0.91 (0.47-1.82) | 0.783 |  |  | 1.24 (0.88-1.79) | 0.231 | 1.19 (0.81-1.76) | 0.382 |

Abbreviations: MA, Migraine with aura; MO, Migraine without aura. OR_adj_ (95% CI) adjusted factors: age, sex, BMI, marital status, nationality, educational level, smoking, alcohol, exercise, history of hypertension, history of diabetes, family history of diabetes, family history of migraine, SAS grade, PHQ9 grade, PSQI grade.

**Table S4.** Associations between the gene polymorphisms of TRP channels and glutamatergic synapse genes and the risk of anxiety and depression in unmatched migraine patients (*P*>0.05).

| SNPs/genotypes/ genetic models | Anxiety No. (%) | | OR_adj_ (95% CI) | *P* | Depression No. (%) | | OR_adj_ (95% CI) | *P* |
| --- | --- | --- | --- | --- | --- | --- | --- | --- |
|  | No Anxiety | Anxiety |  |  | No Depression | Depression |  |  |
| *SLC17A8*-rs11568537 A>C |  |  |  |  |  |  |  |  |
| AA | 141 (69.80) | 32 (65.31) | ref |  | 103 (74.10) | 70 (62.50) | ref |  |
| AC | 55 (27.23) | 16 (32.65) | 1.64 (0.72-3.68) | 0.232 | 32 (23.02) | 39 (34.82) | 1.74 (0.87-3.50) | 0.118 |
| CC | 6 (2.97) | 1 (2.04) | 0.81 (0.04-6.58) | 0.863 | 4 (2.88) | 3 (2.68) | 2.10 (0.28-13.95) | 0.448 |
| Dominant model |  |  | 1.36 (0.17-30.52) | 0.801 |  |  | 0.53 (0.08-3.93) | 0.516 |
| Recessive model |  |  | 1.65 (0.73-3.69) | 0.226 |  |  | 1.71 (0.86-3.42) | 0.129 |
| Additive model |  |  | 0.61 (0.27-1.38) | 0.226 |  |  | 0.59 (0.29-1.17) | 0.129 |
| *GRIK2*-rs2227283 G>A |  |  |  |  |  |  |  |  |
| GG | 80 (39.60) | 23 (46.94) | ref |  | 53 (38.13) | 50 (44.64) | ref |  |
| GA | 103 (50.99) | 21 (42.86) | 0.69 (0.32-1.48) | 0.343 | 74 (53.24) | 50 (44.64) | 0.63 (0.32-1.21) | 0.167 |
| AA | 19 (9.41) | 5 (10.20) | 0.90 (0.22-3.19) | 0.876 | 12 (8.63) | 12 (10.71) | 1.12 (0.36-3.43) | 0.848 |
| Dominant model |  |  | 0.99 (0.36-3.17) | 0.987 |  |  | 1.24 (0.52-3.03) | 0.634 |
| Recessive model |  |  | 0.71 (0.34-1.45) | 0.347 |  |  | 0.62 (0.33-1.15) | 0.128 |
| Additive model |  |  | 1.42 (0.69-2.96) | 0.347 |  |  | 1.62 (0.87-3.06) | 0.128 |
| *SHANK1*-rs3745521 A>G |  |  |  |  |  |  |  |  |
| AA | 64 (31.68) | 16 (32.65) | ref |  | 51 (36.69) | 29 (25.89) | ref |  |
| AG | 92 (45.55) | 22 (44.90) | 1.08 (0.47-2.53) | 0.864 | 62 (44.60) | 52 (46.43) | 1.4 (0.69-2.89) | 0.358 |
| GG | 46 (22.77) | 11 (22.45) | 1.10 (0.40-2.97) | 0.856 | 26 (18.71) | 31 (27.68) | 2.14 (0.92-5.07) | 0.079 |
| Dominant model |  |  | 0.95 (0.41-2.35) | 0.910 |  |  | 0.57 (0.27-1.18) | 0.133 |
| Recessive model |  |  | 1.04 (0.50-2.16) | 0.924 |  |  | 1.01 (0.55-1.86) | 0.978 |
| Additive model |  |  | 0.97 (0.46-2.02) | 0.924 |  |  | 0.99 (0.54-1.83) | 0.978 |
| *SHANK2*-rs3020047 C>T |  |  |  |  |  |  |  |  |
| CC | 146 (72.28) | 36 (73.47) | ref |  | 98 (70.50) | 84 (75.00) | ref |  |
| TC | 53 (26.24) | 12 (24.49) | 0.51 (0.20-1.24) | 0.150 | 39 (28.06) | 26 (23.21) | 0.94 (0.44-2.00) | 0.875 |
| TT | 3 (1.49) | 1 (2.04) | 0.53 (0.02-10.78) | 0.678 | 2 (1.44) | 2 (1.79) | 0.78 (0.05-9.39) | 0.847 |
| Dominant model |  |  | — | — |  |  | 2.41 (0.23-57.13) | 0.498 |
| Recessive model |  |  | 0.53 (0.21-1.26) | 0.161 |  |  | 0.95 (0.45-2.01) | 0.890 |
| Additive model |  |  | 1.90 (0.80-4.85) | 0.161 |  |  | 1.05 (0.50-2.23) | 0.890 |
| *SHANK2*-rs55678639 A>G |  |  |  |  |  |  |  |  |
| AA | 134 (66.34) | 34 (69.39) | ref |  | 91 (65.47) | 77 (68.75) | ref |  |
| AG | 58 (28.71) | 14 (28.57) | 0.79 (0.35-1.73) | 0.571 | 42 (30.22) | 30 (26.79) | 0.84 (0.42-1.64) | 0.602 |
| GG | 10 (4.95) | 1 (2.04) | 0.37 (0.02-2.69) | 0.402 | 6 (4.32) | 5 (4.46) | 0.69 (0.14-3.18) | 0.634 |
| Dominant model |  |  | 2.55 (0.35-54.93) | 0.431 |  |  | 1.37 (0.30-6.57) | 0.683 |
| Recessive model |  |  | 0.83 (0.36-1.80) | 0.638 |  |  | 0.85 (0.44-1.67) | 0.645 |
| Additive model |  |  | 1.21 (0.56-2.78) | 0.638 |  |  | 1.17 (0.60-2.30) | 0.645 |
| *TRPV1*-rs8065080 C>T |  |  |  |  |  |  |  |  |
| CC | 79 (39.11) | 16 (32.65) | ref |  | 57 (41.01) | 38 (33.93) | ref |  |
| TC | 101 (50.00) | 27 (55.10) | 1.54 (0.70-3.52) | 0.289 | 66 (47.48) | 62 (55.36) | 1.18 (0.61-2.28) | 0.627 |
| TT | 22 (10.89) | 6 (12.25) | 1.71 (0.45-6.05) | 0.415 | 16 (11.51) | 12 (10.71) | 0.85 (0.29-2.47) | 0.769 |
| Dominant model |  |  | 0.77 (0.25-2.66) | 0.665 |  |  | 1.31 (0.49-3.57) | 0.597 |
| Recessive model |  |  | 1.36 (0.66-2.86) | 0.411 |  |  | 1.22 (0.67-2.24) | 0.512 |
| Additive model |  |  | 0.74 (0.35-1.52) | 0.411 |  |  | 0.82 (0.45-1.50) | 0.512 |
| *TRPV3*-rs7217270 G>A |  |  |  |  |  |  |  |  |
| GG | 168 (83.17) | 42 (85.71) | ref |  | 114 (82.01) | 96 (85.71) | ref |  |
| AG | 31 (15.35) | 7 (14.29) | 0.94 (0.32-2.48) | 0.906 | 23 (16.55) | 15 (13.39) | 0.64 (0.26-1.52) | 0.315 |
| AA | 3 (1.49) | 0 (0.00) | — | — | 2 (1.44) | 1 (0.89) | 0.17 (0.01-2.68) | 0.226 |
| Dominant model |  |  | 0.77 (0.27-2.00) | 0.608 |  |  | 0.58 (0.24-1.33) | 0.203 |
| Recessive model |  |  | 0.98 (0.34-2.58) | 0.965 |  |  | 0.66 (0.27-1.56) | 0.349 |
| Additive model |  |  | 1.02 (0.39-2.98) | 0.965 |  |  | 1.51 (0.64-3.67) | 0.349 |

OR_adj_ (95% CI) adjusted factors: age, sex, BMI, marital status, nationality, educational level, smoking, alcohol, exercise, history of hypertension, history of diabetes, family history of diabetes, family history of migraine, PSQI grade.

**Table S5.** Associations between the gene polymorphisms of TRP channels and glutamatergic synapse genes and the risk of anxiety and depression in unmatched control subjects.

| SNPs/genotypes/ genetic models | Anxiety No. (%) | | OR_adj_ (95% CI) | *P* | Depression No. (%) | | OR_adj_ (95% CI) | *P* |
| --- | --- | --- | --- | --- | --- | --- | --- | --- |
|  | No Anxiety | Anxiety |  |  | No Depression | Depression |  |  |
| *SHANK2*-rs55678639 A>G |  |  |  |  |  |  |  |  |
| AA | 342 (63.69) | 38 (60.32) | ref |  | 294 (64.33) | 86 (60.14) | ref |  |
| AG | 166 (30.91) | 23 (36.51) | 1.32 (0.72-2.36) | 0.360 | 143 (31.29) | 46 (32.17) | 1.07 (0.67-1.70) | 0.770 |
| GG | 29 (5.40) | 2 (3.18) | 0.90 (0.14-3.35) | 0.893 | 20 (4.38) | 11 (7.69) | 2.46 (1.03-5.71) | **0.038** |
| Dominant model |  |  | 1.22 (0.34-7.85) | 0.796 |  |  | 0.42 (0.18-0.98) | **0.040** |
| Recessive model |  |  | 1.32 (0.73-2.36) | 0.345 |  |  | 0.99 (0.63-1.56) | 0.980 |
| Additive model |  |  | 0.76 (0.42-1.37) | 0.345 |  |  | 1.01 (0.64-1.60) | 0.980 |
| *SLC17A8*-rs11110359 G>A |  |  |  |  |  |  |  |  |
| GG | 417 (77.65) | 45 (71.43) | ref |  | 354 (77.46) | 108 (75.52) | ref |  |
| GA | 107 (19.93) | 15 (23.81) | 1.50 (0.75-2.88) | 0.234 | 91 (19.91) | 31 (21.68) | 1.24 (0.74-2.06) | 0.406 |
| AA | 13 (2.42) | 3 (4.76) | 3.64 (0.75-13.66) | 0.072 | 12 (2.63) | 4 (2.80) | 1.35 (0.33-4.62) | 0.654 |
| Dominant model |  |  | 1.67 (0.87-3.10) | 0.111 |  |  | 1.25 (0.76-2.04) | 0.367 |
| Recessive model |  |  | 1.41 (0.71-2.68) | 0.307 |  |  | 1.23 (0.73-2.04) | 0.428 |
| Additive model |  |  | 0.71 (0.37-1.41) | 0.307 |  |  | 0.81 (0.49-1.37) | 0.428 |
| *SLC17A8*-rs11568537 A>C |  |  |  |  |  |  |  |  |
| AA | 358 (66.67) | 46 (73.02) | ref |  | 308 (67.40) | 96 (67.13) | ref |  |
| AC | 151 (28.12) | 16 (25.40) | 0.90 (0.47-1.66) | 0.740 | 125 (27.35) | 42 (29.37) | 1.21 (0.76-1.92) | 0.413 |
| CC | 28 (5.21) | 1 (1.59) | 0.26 (0.01-1.41) | 0.208 | 24 (5.25) | 5 (3.50) | 0.44 (0.11-1.34) | 0.185 |
| Dominant model |  |  | 3.75 (0.70-70.81) | 0.217 |  |  | 2.42 (0.80-9.50) | 0.154 |
| Recessive model |  |  | 0.95 (0.50-1.75) | 0.874 |  |  | 1.27 (0.80-2.00) | 0.311 |
| Additive model |  |  | 1.05 (0.57-2.02) | 0.874 |  |  | 0.79 (0.50-1.26) | 0.311 |
| *GRIK2*-rs2227283 G>A |  |  |  |  |  |  |  |  |
| GG | 240 (44.69) | 29 (46.03) | ref |  | 203 (44.42) | 66 (46.15) | ref |  |
| GA | 224 (41.71) | 25 (39.68) | 0.87 (0.47-1.59) | 0.647 | 189 (41.36) | 60 (41.96) | 0.84 (0.54-1.32) | 0.455 |
| AA | 73 (13.59) | 9 (14.29) | 1.03 (0.42-2.32) | 0.943 | 65 (14.22) | 17 (11.89) | 0.70 (0.35-1.36) | 0.305 |
| Dominant model |  |  | 0.90 (0.43-2.11) | 0.803 |  |  | 1.31 (0.70-2.57) | 0.409 |
| Recessive model |  |  | 0.86 (0.48-1.51) | 0.607 |  |  | 0.91 (0.60-1.39) | 0.676 |
| Additive model |  |  | 1.16 (0.66-2.07) | 0.607 |  |  | 1.10 (0.72-1.68) | 0.676 |
| *SHANK1*-rs3745521 A>G |  |  |  |  |  |  |  |  |
| AA | 155 (28.86) | 18 (28.57) | ref |  | 136 (29.76) | 37 (25.87) | ref |  |
| AG | 261 (48.60) | 32 (50.79) | 0.96 (0.50-1.87) | 0.894 | 217 (47.48) | 76 (53.15) | 1.18 (0.72-1.96) | 0.515 |
| GG | 121 (22.53) | 13 (20.64) | 0.90 (0.40-1.99) | 0.794 | 104 (22.76) | 30 (20.98) | 0.95 (0.52-1.74) | 0.873 |
| Dominant model |  |  | 1.08 (0.57-2.20) | 0.822 |  |  | 1.17 (0.71-1.97) | 0.543 |
| Recessive model |  |  | 1.00 (0.58-1.75) | 0.990 |  |  | 1.21 (0.80-1.84) | 0.378 |
| Additive model |  |  | 1.00 (0.57-1.73) | 0.990 |  |  | 0.83 (0.55-1.26) | 0.378 |
| *SHANK2*-rs3020047 C>T |  |  |  |  |  |  |  |  |
| CC | 413 (76.91) | 51 (80.95) | ref |  | 359 (78.56) | 105 (73.43) | ref |  |
| TC | 114 (21.23) | 12 (19.05) | 0.74 (0.35-1.45) | 0.399 | 91 (19.91) | 35 (24.48) | 1.15 (0.68-1.89) | 0.599 |
| TT | 10 (1.86) | 0 (0.00) | — | — | 7 (1.53) | 3 (2.10) | 1.29 (0.24-5.62) | 0.743 |
| Dominant model |  |  | 0.68 (0.33-1.33) | 0.285 |  |  | 1.16 (0.70-1.87) | 0.560 |
| Recessive model |  |  | 0.75 (0.35-1.47) | 0.423 |  |  | 1.14 (0.68-1.87) | 0.613 |
| Additive model |  |  | 1.34 (0.68-2.82) | 0.423 |  |  | 0.88 (0.53-1.47) | 0.613 |
| *TRPV1*-rs8065080 C>T |  |  |  |  |  |  |  |  |
| CC | 191 (35.57) | 21 (33.33) | ref |  | 161 (35.23) | 51 (35.66) | ref |  |
| TC | 257 (47.86) | 30 (47.62) | 1.05 (0.56-1.97) | 0.890 | 214 (46.83) | 73 (51.05) | 1.07 (0.68-1.70) | 0.763 |
| TT | 89 (16.57) | 12 (19.05) | 1.18 (0.51-2.62) | 0.689 | 82 (17.94) | 19 (13.29) | 0.57 (0.28-1.10) | 0.099 |
| Dominant model |  |  | 0.87 (0.44-1.86) | 0.704 |  |  | 1.83 (1.01-3.48) | 0.054 |
| Recessive model |  |  | 0.99 (0.57-1.72) | 0.965 |  |  | 1.27 (0.84-1.94) | 0.261 |
| Additive model |  |  | 1.01 (0.58-1.77) | 0.965 |  |  | 0.79 (0.52-1.20) | 0.261 |
| *TRPV1*-rs222741 A>G |  |  |  |  |  |  |  |  |
| AA | 353 (65.74) | 34 (53.97) | ref |  | 291 (63.68) | 96 (67.13) | ref |  |
| GA | 169 (31.47) | 25 (39.68) | 1.60 (0.89-2.86) | 0.112 | 151 (33.04) | 43 (30.07) | 0.79 (0.50-1.24) | 0.317 |
| GG | 15 (2.79) | 4 (6.35) | 2.46 (0.63-7.94) | 0.156 | 15 (3.28) | 4 (2.80) | 0.61 (0.14-2.08) | 0.464 |
| Dominant model |  |  | 0.49 (0.16-1.89) | 0.254 |  |  | 1.51 (0.45-6.40) | 0.538 |
| Recessive model |  |  | 1.49 (0.84-2.62) | 0.167 |  |  | 0.81 (0.51-1.27) | 0.361 |
| Additive model |  |  | 0.67 (0.38-1.19) | 0.167 |  |  | 1.24 (0.79-1.96) | 0.361 |
| *TRPV3*-rs7217270 G>A |  |  |  |  |  |  |  |  |
| GG | 480 (89.39) | 55 (87.30) | ref |  | 407 (89.06) | 128 (89.51) | ref |  |
| AG | 54 (10.06) | 8 (12.70) | 1.29 (0.52-2.90) | 0.556 | 47 (10.28) | 15 (10.49) | 0.68 (0.32-1.39) | 0.310 |
| AA | 3 (0.56) | 0 (0.00) | — | — | 3 (0.66) | 0 (0.00) | — | — |
| Dominant model |  |  | 1.19 (0.48-2.66) | 0.686 |  |  | 0.66 (0.30-1.32) | 0.257 |
| Recessive model |  |  | 1.30 (0.52-2.92) | 0.544 |  |  | 0.69 (0.32-1.39) | 0.316 |
| Additive model |  |  | 0.77 (0.34-1.92) | 0.544 |  |  | 1.46 (0.72-3.16) | 0.316 |
| *TRPV4*-rs3742037 G>A |  |  |  |  |  |  |  |  |
| GG | 365 (67.97) | 39 (61.91) | ref |  | 308 (67.40) | 96 (67.13) | ref |  |
| GA | 155 (28.86) | 20 (31.75) | 0.99 (0.53-1.79) | 0.963 | 135 (29.54) | 40 (27.97) | 0.84 (0.52-1.33) | 0.462 |
| AA | 17 (3.17) | 4 (6.35) | 1.72 (0.44-5.47) | 0.389 | 14 (3.06) | 7 (4.90) | 1.42 (0.49-3.81) | 0.493 |
| Dominant model |  |  | 1.06 (0.59-1.87) | 0.847 |  |  | 0.90 (0.57-1.39) | 0.631 |
| Recessive model |  |  | 0.94 (0.51-1.70) | 0.851 |  |  | 0.82 (0.51-1.29) | 0.399 |
| Additive model |  |  | 1.06 (0.59-1.97) | 0.851 |  |  | 1.22 (0.77-1.96) | 0.399 |
| *TRPM8*-rs17862920 C>T |  |  |  |  |  |  |  |  |
| CC | 287 (53.45) | 34 (53.97) | ref |  | 237 (51.86) | 84 (58.74) | ref |  |
| CT | 211 (39.29) | 26 (41.27) | 1.01 (0.56-1.79) | 0.974 | 185 (40.48) | 52 (36.36) | 0.91 (0.58-1.42) | 0.686 |
| TT | 39 (7.26) | 3 (4.76) | 0.70 (0.16-2.23) | 0.591 | 35 (7.66) | 7 (4.90) | 0.53 (0.19-1.33) | 0.204 |
| Dominant model |  |  | 1.43 (0.46-6.32) | 0.580 |  |  | 1.82 (0.74-5.17) | 0.223 |
| Recessive model |  |  | 1.04 (0.59-1.83) | 0.885 |  |  | 0.97 (0.62-1.49) | 0.879 |
| Additive model |  |  | 0.96 (0.55-1.71) | 0.885 |  |  | 1.03 (0.67-1.61) | 0.879 |
| *TRPM8*-rs10166942 C>T |  |  |  |  |  |  |  |  |
| CC | 224 (41.71) | 29 (46.03) | ref |  | 190 (41.58) | 63 (44.06) | ref |  |
| TC | 251 (46.74) | 30 (47.62) | 0.86 (0.48-1.55) | 0.610 | 218 (47.70) | 63 (44.06) | 0.84 (0.54-1.32) | 0.448 |
| TT | 62 (11.55) | 4 (6.35) | 0.45 (0.13-1.24) | 0.159 | 49 (10.72) | 17 (11.89) | 0.88 (0.43-1.74) | 0.716 |
| Dominant model |  |  | 0.77 (0.44-1.35) | 0.352 |  |  | 0.85 (0.55-1.30) | 0.447 |
| Recessive model |  |  | 0.99 (0.56-1.74) | 0.962 |  |  | 0.86 (0.56-1.32) | 0.499 |
| Additive model |  |  | 1.01 (0.58-1.79) | 0.962 |  |  | 1.16 (0.76-1.78) | 0.499 |
| *TRPM8*-rs7577262 G>A |  |  |  |  |  |  |  |  |
| GG | 197 (36.69) | 23 (36.51) | ref |  | 166 (36.32) | 54 (37.76) | ref |  |
| GA | 262 (48.79) | 32 (50.79) | 1.01 (0.56-1.85) | 0.971 | 224 (49.02) | 70 (48.95) | 1.14 (0.72-1.79) | 0.584 |
| AA | 78 (14.53) | 8 (12.70) | 0.96 (0.37-2.25) | 0.926 | 67 (14.66) | 19 (13.29) | 0.95 (0.47-1.85) | 0.886 |
| Dominant model |  |  | 1.05 (0.48-2.56) | 0.908 |  |  | 1.13 (0.62-2.17) | 0.703 |
| Recessive model |  |  | 1.02 (0.59-1.78) | 0.939 |  |  | 1.15 (0.76-1.75) | 0.514 |
| Additive model |  |  | 0.98 (0.56-1.71) | 0.939 |  |  | 0.87 (0.57-1.32) | 0.514 |

Bold type indicates *P* < 0.05. OR_adj_ (95% CI) adjusted factors: age, sex, BMI, marital status, nationality, educational level, smoking, alcohol, exercise, history of hypertension, history of diabetes, family history of diabetes, family history of migraine, PSQI grade.

**Table S6.** Associations between the gene polymorphisms of TRP channels and glutamatergic synapse genes and the risk of migraine in anxiety or depression patients.

| SNPs/genotypes/ genetic models | Control(N=158)  No. (%) | Migraine(N=123)  No. (%) | OR (95% CI) | *P* | OR_adj_ (95% CI) | *P* |
| --- | --- | --- | --- | --- | --- | --- |
| *TRPM8*-rs7577262 G>A |  |  |  |  |  |  |
| GG | 59 (37.34) | 63 (51.22) | ref |  | ref |  |
| GA | 78 (49.37) | 41 (33.33) | 0.49 (0.29-0.82) | **0.007** | 0.42 (0.24-0.75) | **0.004** |
| AA | 21 (13.29) | 19 (15.45) | 0.85 (0.41-1.73) | 0.650 | 0.97 (0.44-2.15) | 0.946 |
| Dominant model |  |  | 0.84 (0.43-1.65) | 0.608 | 0.69 (0.33-1.44) | 0.319 |
| Recessive model |  |  | 0.51 (0.31-0.83) | **0.007** | 0.43 (0.25-0.73) | **0.002** |
| Additive model |  |  | 1.95 (1.20-3.19) | **0.007** | 2.34 (1.37-4.07) | **0.002** |
| *SLC17A8*-rs11110359 G>A |  |  |  |  |  |  |
| GG | 117 (74.05) | 96 (78.05) | ref |  | ref |  |
| GA | 36 (22.79) | 25 (20.33) | 0.85 (0.47-1.50) | 0.571 | 0.81 (0.42-1.54) | 0.522 |
| AA | 5 (3.17) | 2 (1.63) | 0.49 (0.07-2.32) | 0.397 | 0.86 (0.12-4.34) | 0.858 |
| Dominant model |  |  | 0.80 (0.46-1.39) | 0.438 | 0.82 (0.44-1.50) | 0.515 |
| Recessive model |  |  | 0.87 (0.48-1.53) | 0.620 | 0.81 (0.42-1.54) | 0.530 |
| Additive model |  |  | 1.16 (0.65-2.07) | 0.620 | 1.23 (0.65-2.36) | 0.530 |
| *SLC17A8*-rs11568537 A>C |  |  |  |  |  |  |
| AA | 107 (67.72) | 77 (62.60) | ref |  | ref |  |
| AC | 46 (29.11) | 43 (34.96) | 1.30 (0.78-2.16) | 0.313 | 1.42 (0.81-2.53) | 0.225 |
| CC | 5 (3.17) | 3 (2.44) | 0.83 (0.17-3.50) | 0.807 | 0.74 (0.14-3.46) | 0.702 |
| Dominant model |  |  | 1.31 (0.31-6.48) | 0.718 | 1.52 (0.33-8.18) | 0.595 |
| Recessive model |  |  | 1.31 (0.79-2.17) | 0.297 | 1.44 (0.82-2.55) | 0.204 |
| Additive model |  |  | 0.76 (0.46-1.27) | 0.297 | 0.69 (0.39-1.22) | 0.204 |
| *GRIK2*-rs2227283 G>A |  |  |  |  |  |  |
| GG | 74 (46.84) | 53 (43.09) | ref |  | ref |  |
| GA | 66 (41.77) | 56 (45.53) | 1.19 (0.72-1.96) | 0.508 | 1.13 (0.65-1.99) | 0.662 |
| AA | 18 (11.39) | 14 (11.38) | 1.09 (0.49-2.37) | 0.836 | 0.80 (0.32-1.93) | 0.617 |
| Dominant model |  |  | 1.00 (0.48-2.134) | 0.998 | 1.33 (0.57-3.18) | 0.511 |
| Recessive model |  |  | 1.17 (0.72-1.88) | 0.529 | 1.18 (0.69-2.03) | 0.541 |
| Additive model |  |  | 0.86 (0.53-1.38) | 0.529 | 0.85 (0.49-1.45) | 0.541 |
| *SHANK1*-rs3745521 A>G |  |  |  |  |  |  |
| AA | 42 (26.58) | 33 (26.83) | ref |  | ref |  |
| AG | 83 (52.53) | 57 (46.34) | 0.87 (0.50-1.55) | 0.642 | 0.90 (0.48-1.68) | 0.729 |
| GG | 33 (20.89) | 33 (26.83) | 1.27 (0.66-2.48) | 0.476 | 1.37 (0.65-2.90) | 0.411 |
| Dominant model |  |  | 0.72 (0.41-1.25) | 0.245 | 0.68 (0.37-1.26) | 0.218 |
| Recessive model |  |  | 0.78 (0.49-1.25) | 0.304 | 0.77 (0.46-1.30) | 0.327 |
| Additive model |  |  | 1.28 (0.80-2.06) | 0.304 | 1.30 (0.77-2.19) | 0.327 |
| *SHANK2*-rs3020047 C>T |  |  |  |  |  |  |
| CC | 116 (73.42) | 88 (71.55) | ref |  | ref |  |
| TC | 39 (24.68) | 32 (26.02) | 1.08 (0.63-1.86) | 0.777 | 0.94 (0.51-1.73) | 0.845 |
| TT | 3 (1.90) | 3 (2.44) | 1.32 (0.24-7.27) | 0.739 | 0.78 (0.13-4.68) | 0.779 |
| Dominant model |  |  | 1.10 (0.65-1.86) | 0.727 | 0.93 (0.51-1.66) | 0.795 |
| Recessive model |  |  | 1.07 (0.62-1.84) | 0.799 | 0.95 (0.51-1.74) | 0.861 |
| Additive model |  |  | 0.93 (0.54-1.61) | 0.799 | 1.06 (0.58-1.95) | 0.861 |
| *SHANK2*-rs55678639 A>G |  |  |  |  |  |  |
| AA | 97 (61.39) | 84 (68.293) | ref |  | ref |  |
| AG | 50 (31.65) | 34 (27.642) | 0.79 (0.46-1.32) | 0.366 | 0.64 (0.35-1.15) | 0.139 |
| GG | 11 (6.96) | 5 (4.065) | 0.53 (0.16-1.51) | 0.249 | 0.37 (0.10-1.20) | 0.110 |
| Dominant model |  |  | 1.77 (0.62-5.73) | 0.304 | 2.32 (0.73-8.35) | 0.169 |
| Recessive model |  |  | 0.83 (0.49-1.38) | 0.467 | 0.70 (0.39-1.24) | 0.224 |
| Additive model |  |  | 1.21 (0.72-2.05) | 0.467 | 1.43 (0.81-2.57) | 0.224 |
| *TRPV1*-rs8065080 C>T |  |  |  |  |  |  |
| CC | 55 (34.81) | 43 (34.96) | ref |  | ref |  |
| TC | 78 (49.37) | 68 (55.29) | 1.12 (0.67-1.87) | 0.678 | 1.14 (0.64-2.03) | 0.661 |
| TT | 25 (15.82) | 12 (9.76) | 0.61 (0.27-1.34) | 0.229 | 0.53 (0.21-1.30) | 0.173 |
| Dominant model |  |  | 1.74 (0.85-3.73) | 0.139 | 2.03 (0.91-4.80) | 0.093 |
| Recessive model |  |  | 1.27 (0.79-2.04) | 0.325 | 1.34 (0.80-2.28) | 0.269 |
| Additive model |  |  | 0.79 (0.49-1.26) | 0.325 | 0.74 (0.44-1.26) | 0.269 |
| *TRPV1*-rs222741 A>G |  |  |  |  |  |  |
| AA | 101 (63.92) | 71 (57.72) | ref |  | ref |  |
| GA | 51 (32.28) | 48 (39.02) | 1.34 (0.81-2.21) | 0.250 | 1.28 (0.74-2.22) | 0.376 |
| GG | 6 (3.80) | 4 (3.25) | 0.95 (0.24-3.44) | 0.936 | 1.14 (0.25-4.67) | 0.862 |
| Dominant model |  |  | 1.17 (0.33-4.68) | 0.807 | 0.96 (0.24-4.36) | 0.952 |
| Recessive model |  |  | 1.34 (0.82-2.20) | 0.241 | 1.27 (0.74-2.20) | 0.384 |
| Additive model |  |  | 0.75 (0.46-1.22) | 0.241 | 0.79 (0.46-1.36) | 0.384 |
| *TRPV3*-rs7217270 G>A |  |  |  |  |  |  |
| GG | 141 (89.24) | 105 (85.37) | ref |  | ref |  |
| AG | 17 (10.76) | 17 (13.82) | 1.34 (0.65-2.77) | 0.421 | 1.33 (0.60-2.93) | 0.481 |
| AA | 0 (0.00) | 1 (0.81) | — | — | — | — |
| Dominant model |  |  | 1.42 (0.70-2.91) | 0.331 | 1.40 (0.64-3.05) | 0.401 |
| Recessive model |  |  | 1.33 (0.65-2.74) | 0.436 | 1.32 (0.60-2.92) | 0.487 |
| Additive model |  |  | 0.75 (0.37-1.55) | 0.436 | 0.76 (0.34-1.67) | 0.487 |
| *TRPV4*-rs3742037 G>A |  |  |  |  |  |  |
| GG | 107 (67.72) | 76 (61.79) | ref |  | ref |  |
| GA | 44 (27.85) | 41 (33.33) | 1.31 (0.78-2.20) | 0.304 | 1.35 (0.75-2.42) | 0.313 |
| AA | 7 (4.43) | 6 (4.88) | 1.21 (0.38-3.77) | 0.744 | 1.36 (0.37-4.86) | 0.630 |
| Dominant model |  |  | 1.30 (0.79-2.13) | 0.301 | 1.35 (0.78-2.36) | 0.286 |
| Recessive model |  |  | 1.30 (0.78-2.16) | 0.321 | 1.32 (0.74-2.36) | 0.340 |
| Additive model |  |  | 0.77 (0.46-1.29) | 0.321 | 0.76 (0.42-1.35) | 0.340 |
| *TRPM8*-rs17862920 C>T |  |  |  |  |  |  |
| CC | 90 (56.96) | 79 (64.23) | ref |  | ref |  |
| CT | 61 (38.61) | 37 (30.08) | 0.69 (0.41-1.15) | 0.154 | 0.70 (0.39-1.23) | 0.218 |
| TT | 7 (4.430) | 7 (5.69) | 1.14 (0.38-3.46) | 0.815 | 1.64 (0.48-5.63) | 0.427 |
| Dominant model |  |  | 0.77 (0.26-2.30) | 0.631 | 0.54 (0.16-1.81) | 0.310 |
| Recessive model |  |  | 0.68 (0.41-1.13) | 0.138 | 0.67 (0.38-1.18) | 0.167 |
| Additive model |  |  | 1.46 (0.89-2.43) | 0.138 | 1.49 (0.85-2.64) | 0.167 |
| *TRPM8*-rs10166942 C>T |  |  |  |  |  |  |
| CC | 70 (44.30) | 52 (42.28) | ref |  | ref |  |
| TC | 71 (44.94) | 51 (41.46) | 0.97 (0.58-1.61) | 0.897 | 0.96 (0.55-1.69) | 0.891 |
| TT | 17 (10.76) | 20 (16.26) | 1.58 (0.76-3.35) | 0.223 | 1.71 (0.76-3.87) | 0.193 |
| Dominant model |  |  | 1.09 (0.68-1.75) | 0.734 | 1.11 (0.66-1.88) | 0.693 |
| Recessive model |  |  | 0.87 (0.54-1.40) | 0.560 | 0.85 (0.50-1.45) | 0.552 |
| Additive model |  |  | 1.15 (0.72-1.86) | 0.560 | 1.18 (0.69-2.01) | 0.552 |

Bold type indicates *P* < 0.05. OR_adj_ (95% CI) adjusted factors: age, sex, BMI, marital status, nationality, educational level, smoking, alcohol, exercise, history of hypertension, history of diabetes, family history of diabetes, family history of migraine, PSQI grade.

**Table S7.** Basic demographic and clinical characteristics of the migraine, MA, MO and control groups in matched subjects.

| Characteristics | Control | Migraine | MA | MO | *P*_a_ | *P*_b_ | *P*_c_ | *P*_d_ |
| --- | --- | --- | --- | --- | --- | --- | --- | --- |
|  | N=452 | N=226 | N=46 | N=180 |  |  |  |  |
| Age, Mean ± SD | 42.75 ±13.48 | 42.39 ±13.49 | 41.70±12.46 | 42.57±13.77 | 0.743 | 0.588 | 0.880 | 0.678 |
| Age grade (%) |  |  |  |  | 0.942 | 0.781 | 0.987 | 0.844 |
| ≤35 | 198 (43.81) | 99 (43.81) | 21 (45.65) | 78 (43.33) |  |  |  |  |
| 35~ | 106 (23.45) | 54 (23.89) | 10 (21.74) | 44 (24.44) |  |  |  |  |
| 50~ | 89 (19.69) | 47 (20.80) | 11 (23.91) | 36 (20.00) |  |  |  |  |
| 60~ | 59 (13.05) | 26 (11.50) | 4 (8.70) | 22 (12.22) |  |  |  |  |
| Sex (%) |  |  |  |  | 1.000 | 1.000 | 1.000 | 1.000 |
| Male | 92 (20.35) | 46 (20.35) | 9 (19.57) | 37 (20.56) |  |  |  |  |
| Female | 360 (79.65) | 180 (79.65) | 37 (80.44) | 143 (79.44) |  |  |  |  |
| BMI (kg/m2), Mean ± SD | 23.62 ±3.38 | 23.15±3.21 | 22.71 ±2.69 | 23.27 ±3.33 | 0.082 | **0.038** | 0.234 | 0.240 |
| Marital status (%) |  |  |  |  | 0.229 | 0.443 | 0.325 | 0.896 |
| Unmarried | 179 (39.60) | 78 (34.51) | 15 (32.61) | 63 (35.00) |  |  |  |  |
| Married | 273 (60.40) | 148 (65.49) | 31 (67.39) | 117 (65.00) |  |  |  |  |
| Nationality (%) |  |  |  |  | 0.787 | 0.061 | 0.892 | 0.079 |
| Han nationality | 420 (92.92) | 212 (93.81) | 46 (100.00) | 166 (92.22) |  |  |  |  |
| Minority | 32 (7.08) | 14 (6.20) | 0 (0.00) | 14 (7.78) |  |  |  |  |
| Educational level (%) |  |  |  |  | **<0.001** | 0.086 | **<0.001** | 0.218 |
| Elementary and below | 18 (3.98) | 24 (10.62) | 6 (13.04) | 18 (10.00) |  |  |  |  |
| Junior school | 48 (10.62) | 49 (21.68) | 5 (10.87) | 44 (24.44) |  |  |  |  |
| Senior school | 81 (17.92) | 29 (12.83) | 7 (15.22) | 22 (12.22) |  |  |  |  |
| College and above | 305 (67.48) | 124 (54.87) | 28 (60.87) | 96 (53.33) |  |  |  |  |
| Smoking (%) |  |  |  |  | 0.344 | 1.000 | 0.307 | 0.848 |
| Yes | 64 (14.16) | 39 (17.26) | 7 (15.22) | 32 (17.78) |  |  |  |  |
| No | 388 (85.84) | 187 (82.74) | 39 (84.78) | 148 (82.22) |  |  |  |  |
| Alcohol (%) |  |  |  |  | 0.744 | 0.885 | 0.819 | 1.000 |
| Yes | 78 (17.26) | 36 (15.93) | 7 (15.22) | 29 (16.11) |  |  |  |  |
| No | 374 (82.74) | 190 (84.07) | 39 (84.78) | 151 (83.89) |  |  |  |  |
| Exercise (%) |  |  |  |  | **0.017** | 0.320 | **0.025** | 1.000 |
| Yes | 217 (48.01) | 86 (38.05) | 18 (39.13) | 68 (37.78) |  |  |  |  |
| No | 235 (51.99) | 140 (61.95) | 28 (60.87) | 112 (62.22) |  |  |  |  |
| History of hypertension (%) |  |  |  |  | 0.413 | 0.101 | 0.825 | 0.271 |
| Yes | 36 (7.97) | 23 (10.18) | 7 (15.22) | 16 (8.89) |  |  |  |  |
| No | 416 (92.04) | 203 (89.82) | 39 (84.78) | 164 (91.11) |  |  |  |  |
| History of diabetes (%) |  |  |  |  | 0.322 | 0.715 | 0.459 | 1.000 |
| Yes | 23 (5.09) | 7 (3.10) | 1 (2.17) | 6 (3.33) |  |  |  |  |
| No | 429 (94.91) | 219 (96.90) | 45 (97.83) | 174 (96.67) |  |  |  |  |
| Family history of diabetes (%) |  |  |  |  | 0.161 | 0.397 | 0.238 | 0.955 |
| Yes | 71 (15.71) | 46 (20.35) | 10 (21.74) | 36 (20.00) |  |  |  |  |
| No | 381 (84.29) | 180 (79.65) | 36 (78.26) | 144 (80.00) |  |  |  |  |
| Family history of migraine (%) |  |  |  |  | **<0.001** | **0.031** | **<0.001** | 1.000 |
| Yes | 90 (19.91) | 77 (34.07) | 16 (34.78) | 61 (33.89) |  |  |  |  |
| No | 362 (80.09) | 149 (65.93) | 30 (65.22) | 119 (66.11) |  |  |  |  |
| SAS score, Mean ± SD | 37.18 ±9.69 | 36.54 ±11.58 | 39.12 ±13.80 | 35.88 ±10.88 | 0.472 | 0.357 | 0.162 | 0.144 |
| SAS grade (%) |  |  |  |  | **0.013** | **0.001** | 0.156 | **0.039** |
| No anxiety | 398 (88.05) | 180 (79.65) | 31 (67.39) | 149 (82.78) |  |  |  |  |
| Mild anxiety | 44 (9.74) | 39 (17.26) | 12 (26.09) | 27 (15.00) |  |  |  |  |
| Moderate anxiety | 10 (2.21) | 7 (3.10) | 3 (6.52) | 4 (2.22) |  |  |  |  |
| PHQ-9 score, Mean ± SD | 2.92 ±4.00 | 4.01 ±4.21 | 5.04 ±4.38 | 3.74 ±4.14 | **0.001** | **0.003** | **0.023** | 0.074 |
| PHQ-9 grade (%) |  |  |  |  | **<0.001** | **<0.001** | **0.002** | 0.071 |
| No depression | 330 (73.01) | 125 (55.31) | 18 (39.13) | 107 (59.44) |  |  |  |  |
| Mild depression | 70 (15.49) | 70 (30.97) | 19 (41.30) | 51 (28.33) |  |  |  |  |
| Moderate depression | 39 (8.63) | 21 (9.29) | 6 (13.04) | 15 (8.33) |  |  |  |  |
| Moderate to severe depression | 13 (2.88) | 10 (4.43) | 3 (6.52) | 7 (3.89) |  |  |  |  |
| PSQI score, Mean ± SD | 3.92 ±2.65 | 4.86 ±2.84 | 5.65 ±3.15 | 4.66 ±2.73 | **<0.001** | **0.001** | **0.002** | 0.055 |
| PSQI grade (%) |  |  |  |  | **<0.001** | **0.002** | **0.002** | 0.276 |
| Good sleep quality | 295 (65.27) | 112 (49.56) | 19 (41.30) | 93 (51.67) |  |  |  |  |
| Poor sleep quality | 157 (34.74) | 114 (50.44) | 27 (58.70) | 87 (48.33) |  |  |  |  |

Abbreviations: MA, Migraine with aura; MO, Migraine without aura; BMI, Body Mass Index; SAS, the Self-Rating Anxiety Scale; PHQ-9, the Patient Health Questionnaire-9; PSQI, the Pittsburgh Sleep Quality Index. *P*_a_, comparison of Migraine with Controls; *P*_b_, comparison of MA with Controls; *P*_c_, comparison of MO with Controls; *P*_d_, comparison of MA with MO. *P* < 0.05 are considered statistically significant, and shown in bold.

**Table S8.** Associations between the gene polymorphisms of TRP channels and glutamatergic synapse genes and the risk of migraine in matched subjects.

| SNPs/genotypes/  genetic models | Control  No. (%) | Migraine  No. (%) | OR (95% CI) | *P* | OR_adj_ (95% CI) | *P* |
| --- | --- | --- | --- | --- | --- | --- |
| *TRPV3*-rs7217270 G>A | |  |  |  |  |  |
| GG | 404 (89.38) | 188 (83.19) | ref |  | ref |  |
| AG | 46 (10.18) | 35 (15.49) | 1.64 (1.01-2.62) | **0.041** | 1.57 (0.93-2.62) | 0.085 |
| AA | 2 (0.44) | 3 (1.33) | 3.22 (0.53-24.63) | 0.202 | 2.50 (0.38-20.26) | 0.339 |
| Dominant model |  |  | 1.70 (1.07-2.69) | **0.023** | 1.62 (0.98-2.66) | 0.058 |
| Recessive model |  |  | 1.62 (1.00-2.59) | **0.046** | 1.56 (0.93-2.61) | 0.090 |
| Additive model |  |  | 0.62 (0.39-1.00) | **0.046** | 0.64 (0.38-1.08) | 0.090 |
| *TRPM8*-rs17862920 C>T | |  |  |  |  |  |
| CC | 243 (53.76) | 143 (63.27) | ref |  | ref |  |
| CT | 177 (39.16) | 72 (31.86) | 0.69 (0.49-0.97) | **0.035** | 0.73 (0.50-1.07) | 0.105 |
| TT | 32 (7.08) | 11 (4.87) | 0.58 (0.27-1.16) | 0.141 | 0.57 (0.25-1.22) | 0.167 |
| Dominant model |  |  | 1.49 (0.76-3.15) | 0.268 | 1.54 (0.73-3.50) | 0.273 |
| Recessive model |  |  | 0.73 (0.52-1.02) | 0.063 | 0.77 (0.53-1.12) | 0.171 |
| Additive model |  |  | 1.38 (0.99-1.94) | 0.063 | 1.29 (0.90-1.88) | 0.171 |
| *TRPM8*-rs7577262 G>A | |  |  |  |  |  |
| GG | 169 (37.39) | 106 (46.90) | ref |  | ref |  |
| GA | 221 (48.89) | 94 (41.59) | 0.68 (0.48-0.95) | **0.026** | 0.74 (0.51-1.07) | 0.110 |
| AA | 62 (13.72) | 26 (11.50) | 0.67 (0.39-1.11) | 0.128 | 0.66 (0.37-1.15) | 0.152 |
| Dominant model |  |  | 1.22 (0.76-2.02) | 0.420 | 1.29 (0.77-2.23) | 0.341 |
| Recessive model |  |  | 0.74 (0.54-1.03) | 0.073 | 0.81 (0.57-1.15) | 0.241 |
| Additive model |  |  | 1.34 (0.97-1.86) | 0.073 | 1.23 (0.87-1.75) | 0.241 |
| *TRPM8*-rs10166942 C>T | |  |  |  |  |  |
| CC | 185 (40.93) | 82 (36.28) | ref |  | ref |  |
| TC | 219 (48.45) | 110 (48.67) | 1.13 (0.80-1.61) | 0.479 | 1.26 (0.86-1.84) | 0.233 |
| TT | 48 (10.62) | 34 (15.04) | 1.60 (0.96-2.66) | 0.072 | 1.59 (0.91-2.76) | 0.102 |
| Dominant model |  |  | 1.22 (0.88-1.70) | 0.243 | 1.32 (0.93-1.90) | 0.127 |
| Recessive model |  |  | 1.01 (0.73-1.39) | 0.957 | 1.12 (0.79-1.59) | 0.521 |
| Additive model |  |  | 0.99 (0.72-1.37) | 0.957 | 0.89 (0.63-1.26) | 0.521 |
| *SLC17A8*-rs11110359 G>A | |  |  |  |  |  |
| GG | 350 (77.43) | 165 (73.01) | ref |  | ref |  |
| GA | 86 (19.03) | 56 (24.78) | 1.38 (0.94-2.02) | 0.099 | 1.37 (0.90-2.08) | 0.145 |
| AA | 16 (3.54) | 5 (2.21) | 0.66 (0.21-1.73) | 0.430 | 0.68 (0.21-1.88) | 0.480 |
| Dominant model |  |  | 1.27 (0.88-1.83) | 0.204 | 1.26 (0.84-1.88) | 0.262 |
| Recessive model |  |  | 1.40 (0.95-2.05) | 0.083 | 1.39 (0.91-2.11) | 0.124 |
| Additive model |  |  | 0.71 (0.49-1.05) | 0.083 | 0.72 (0.48-1.10) | 0.124 |
| *SLC17A8*-rs11568537 A>C | |  |  |  |  |  |
| AA | 299 (66.15) | 156 (69.03) | ref |  | ref |  |
| AC | 132 (29.20) | 63 (27.88) | 0.92 (0.64-1.30) | 0.625 | 0.89 (0.60-1.31) | 0.557 |
| CC | 21 (4.65) | 7 (3.10) | 0.64 (0.25-1.47) | 0.317 | 0.55 (0.20-1.37) | 0.221 |
| Dominant model |  |  | 1.52 (0.67-3.92) | 0.343 | 1.76 (0.71-4.86) | 0.244 |
| Recessive model |  |  | 0.94 (0.66-1.33) | 0.719 | 0.92 (0.62-1.35) | 0.660 |
| Additive model |  |  | 1.07 (0.75-1.53) | 0.719 | 1.09 (0.74-1.61) | 0.660 |
| *GRIK2*-rs2227283 G>A | |  |  |  |  |  |
| GG | 208 (46.02) | 93 (41.15) | ref |  | ref |  |
| GA | 188 (41.59) | 111 (49.12) | 1.32 (0.94-1.86) | 0.108 | 1.29 (0.89-1.86) | 0.182 |
| AA | 56 (12.39) | 22 (9.74) | 0.88 (0.50-1.51) | 0.645 | 0.75 (0.40-1.36) | 0.356 |
| Dominant model |  |  | 1.31 (0.79-2.25) | 0.308 | 1.51 (0.86-2.74) | 0.159 |
| Recessive model |  |  | 1.36 (0.98-1.87) | 0.063 | 1.36 (0.96-1.93) | 0.085 |
| Additive model |  |  | 0.74 (0.54-1.02) | 0.063 | 0.74 (0.52-1.04) | 0.085 |
| *TRPV1*-rs222741 A>G |  |  |  |  |  |  |
| AA | 291 (64.38) | 146 (64.60) | ref |  | ref |  |
| GA | 146 (32.30) | 73 (32.30) | 1.00 (0.71-1.40) | 0.984 | 0.90 (0.62-1.31) | 0.591 |
| GG | 15 (3.32) | 7 (3.10) | 0.93 (0.35-2.26) | 0.877 | 0.85 (0.30-2.21) | 0.752 |
| Dominant model |  |  | 1.07 (0.45-2.85) | 0.878 | 1.13 (0.44-3.16) | 0.807 |
| Recessive model |  |  | 1.00 (0.71-1.40) | 1.000 | 0.91 (0.62-1.32) | 0.618 |
| Additive model |  |  | 1.00 (0.71-1.41) | 1.000 | 1.10 (0.76-1.61) | 0.618 |
| *SHANK2*-rs3020047 C>T | |  |  |  |  |  |
| CC | 342 (75.66) | 161 (71.24) | ref |  | ref |  |
| TC | 102 (22.57) | 61 (26.99) | 1.27 (0.88-1.83) | 0.203 | 1.24 (0.83-1.87) | 0.294 |
| TT | 8 (1.77) | 4 (1.77) | 1.06 (0.28-3.42) | 0.923 | 1.01 (0.25-3.59) | 0.987 |
| Dominant model |  |  | 1.26 (0.87-1.79) | 0.215 | 1.23 (0.82-1.82) | 0.315 |
| Recessive model |  |  | 1.27 (0.88-1.83) | 0.204 | 1.24 (0.83-1.86) | 0.293 |
| Additive model |  |  | 0.79 (0.55-1.142) | 0.204 | 0.80 (0.54-1.21) | 0.293 |
| *TRPV4*-rs3742037 G>A | |  |  |  |  |  |
| GG | 297 (65.71) | 153 (67.70) | ref |  | ref |  |
| GA | 141 (31.20) | 64 (28.32) | 0.88 (0.62-1.25) | 0.483 | 0.83 (0.56-1.21) | 0.337 |
| AA | 14 (3.10) | 9 (3.98) | 1.25 (0.51-2.91) | 0.614 | 1.06 (0.40-2.70) | 0.898 |
| Dominant model |  |  | 0.91 (0.65-1.28) | 0.605 | 0.85 (0.59-1.23) | 0.390 |
| Recessive model |  |  | 0.87 (0.61-1.23) | 0.442 | 0.83 (0.56-1.21) | 0.326 |
| Additive model |  |  | 1.15 (0.81-1.64) | 0.442 | 1.21 (0.83-1.78) | 0.326 |
| *SHANK1*-rs3745521 A>G | |  |  |  |  |  |
| AA | 130 (28.76) | 72 (31.86) | ref |  | ref |  |
| AG | 219 (48.45) | 102 (45.13) | 0.84 (0.58-1.22) | 0.361 | 0.77 (0.52-1.16) | 0.211 |
| GG | 103 (22.79) | 52 (23.01) | 0.91 (0.59-1.41) | 0.680 | 0.81 (0.50-1.31) | 0.385 |
| Dominant model |  |  | 0.99 (0.68-1.45) | 0.948 | 1.06 (0.70-1.61) | 0.800 |
| Recessive model |  |  | 0.88 (0.64-1.21) | 0.415 | 0.85 (0.60-1.20) | 0.352 |
| Additive model |  |  | 1.14 (0.83-1.58) | 0.415 | 1.18 (0.83-1.67) | 0.352 |
| *SHANK2*-rs55678639 A>G | |  |  |  |  |  |
| AA | 284 (62.83) | 150 (66.37) | ref |  | ref |  |
| AG | 146 (32.30) | 65 (28.76) | 0.84 (0.59-1.20) | 0.343 | 0.83 (0.57-1.22) | 0.347 |
| GG | 22 (4.87) | 11 (4.87) | 0.95 (0.43-1.97) | 0.886 | 0.82 (0.35-1.82) | 0.636 |
| Dominant model |  |  | 1.00 (0.49-2.18) | 1.000 | 1.15 (0.53-2.68) | 0.732 |
| Recessive model |  |  | 0.85 (0.60-1.20) | 0.348 | 0.84 (0.58-1.23) | 0.378 |
| Additive model |  |  | 1.18 (0.84-1.68) | 0.348 | 1.19 (0.81-1.74) | 0.378 |
| *TRPV1*-rs8065080 C>T | |  |  |  |  |  |
| CC | 158 (34.96) | 87 (38.50) | ref |  | ref |  |
| TC | 216 (47.79) | 112 (49.56) | 0.94 (0.67-1.33) | 0.734 | 0.91 (0.62-1.33) | 0.621 |
| TT | 78 (17.26) | 27 (11.95) | 0.63 (0.37-1.04) | 0.074 | 0.59 (0.33-1.01) | 0.060 |
| Dominant model |  |  | 1.54 (0.97-2.50) | 0.073 | 1.61 (0.98-2.73) | 0.067 |
| Recessive model |  |  | 1.07 (0.78-1.48) | 0.664 | 1.06 (0.75-1.50) | 0.742 |
| Additive model |  |  | 0.93 (0.68-1.28) | 0.664 | 0.94 (0.67-1.34) | 0.742 |

Bold type indicates *P* < 0.05. OR_adj_ (95% CI) adjusted factors: age, sex, BMI, marital status, nationality, educational level, smoking, alcohol, exercise, history of hypertension, history of diabetes, family history of diabetes, family history of migraine, SAS grade, PHQ9 grade, PSQI grade.

**Table S9.** Associations between the gene polymorphisms of TRP channels and glutamatergic synapse genes and the risk of migraine by aura in matched subjects.

| SNPs/genotypes/  genetic models | Control  No. (%) | MA | | | | |  | MO | | | | |
| --- | --- | --- | --- | --- | --- | --- | --- | --- | --- | --- | --- | --- |
|  |  | No. (%) | OR (95%CI) | *P* | OR_adj_(95%CI) | *P* |  | No. (%) | OR (95%CI) | *P* | OR_adj_(95%CI) | *P* |
| *SLC17A8*-rs11110359 G>A | |  |  |  |  |  |  |  |  |  |  |  |
| GG | 350 (77.43) | 38 (82.61) | ref |  | ref |  |  | 127 (70.56) | ref |  | ref |  |
| GA | 86 (19.03) | 8 (17.39) | 0.86 (0.36-1.81) | 0.704 | 0.78 (0.30-1.82) | 0.589 |  | 48 (26.67) | 1.54 (1.02-2.31) | **0.038** | 1.62 (1.03-2.54) | **0.034** |
| AA | 16 (3.54) | 0 (0.00) | — | — | — | — |  | 5 (2.78) | 0.86 (0.28-2.25) | 0.775 | 0.88 (0.26-2.47) | 0.813 |
| Dominant model |  |  | 0.72 (0.31-1.52) | 0.422 | 0.67 (0.26-1.54) | 0.370 |  |  | 1.43 (0.97-2.11) | 0.070 | 1.50 (0.98-2.30) | 0.062 |
| Recessive model |  |  | 0.90 (0.38-1.90) | 0.787 | 0.83 (0.32-1.92) | 0.679 |  |  | 1.55 (1.03-2.31) | **0.035** | 1.63 (1.04-2.54) | **0.031** |
| Additive model |  |  | 1.12 (0.53-2.65) | 0.787 | 1.21 (0.52-3.12) | 0.679 |  |  | 0.65 (0.43-0.97) | **0.035** | 0.61 (0.39-0.96) | **0.031** |
| *SLC17A8*-rs11568537 A>C | |  |  |  |  |  |  |  |  |  |  |  |
| AA | 299 (66.150) | 28 (60.870) | ref |  | ref |  |  | 128 (71.11) | ref |  | ref |  |
| AC | 132 (29.204) | 14 (30.435) | 1.13 (0.56-2.19) | 0.717 | 1.07 (0.49-2.27) | 0.863 |  | 49 (27.22) | 0.87 (0.59-1.27) | 0.471 | 0.84 (0.55-1.27) | 0.413 |
| CC | 21 (4.646) | 4 (8.696) | 2.03 (0.56-5.81) | 0.221 | 2.04 (0.48-7.09) | 0.294 |  | 3 (1.67) | 0.33 (0.08-0.99) | 0.080 | 0.23 (0.05-0.76) | **0.030** |
| Dominant model |  |  | 0.51 (0.18-1.82) | 0.239 | 0.50 (0.15-2.11) | 0.301 |  |  | 2.88 (0.98-12.28) | 0.090 | 4.09 (1.26-18.67) | **0.035** |
| Recessive model |  |  | 1.06 (0.53-2.01) | 0.861 | 1.01 (0.46-2.14) | 0.981 |  |  | 0.91 (0.61-1.33) | 0.619 | 0.89 (0.59-1.35) | 0.590 |
| Additive model |  |  | 0.94 (0.50-1.88) | 0.861 | 0.99 (0.48-2.16) | 0.981 |  |  | 1.10 (0.75-1.63) | 0.619 | 1.12 (0.74-1.71) | 0.590 |
| *GRIK2*-rs2227283 G>A | |  |  |  |  |  |  |  |  |  |  |  |
| GG | 208 (46.02) | 14 (30.44) | ref |  | ref |  |  | 79 (43.89) | ref |  | ref |  |
| GA | 188 (41.59) | 27 (58.70) | 2.13 (1.10-4.30) | **0.028** | 2.15 (1.03-4.65) | **0.045** |  | 84 (46.67) | 1.18 (0.82-1.70) | 0.383 | 1.15 (0.77-1.71) | 0.492 |
| AA | 56 (12.39) | 5 (10.87) | 1.33 (0.41-3.63) | 0.602 | 1.22 (0.34-3.82) | 0.741 |  | 17 (9.44) | 0.80 (0.43-1.43) | 0.465 | 0.70 (0.35-1.33) | 0.289 |
| Dominant model |  |  | 1.16 (0.48-3.47) | 0.765 | 1.26 (0.46-4.24) | 0.679 |  |  | 1.36 (0.78-2.47) | 0.297 | 1.53 (0.83-2.95) | 0.185 |
| Recessive model |  |  | 2.00 (1.08-3.74) | **0.028** | 2.05 (1.04-4.15) | **0.041** |  |  | 1.23 (0.87-1.74) | 0.245 | 1.231 (0.84-1.80) | 0.283 |
| Additive model |  |  | 0.50 (0.27-0.92) | **0.028** | 0.49 (0.24-0.97) | **0.041** |  |  | 0.81 (0.575-1.15) | 0.245 | 0.812 (0.56-1.19) | 0.283 |
| *TRPV3*-rs7217270 G>A | |  |  |  |  |  |  |  |  |  |  |  |
| GG | 404 (89.38) | 40 (86.96) | ref |  | ref |  |  | 148 (82.22) | ref |  | ref |  |
| AG | 46 (10.18) | 6 (13.04) | 1.32 (0.48-3.07) | 0.553 | 0.92 (0.30-2.48) | 0.883 |  | 29 (16.11) | 1.72 (1.03-2.83) | **0.034** | 1.62 (0.93-2.77) | 0.084 |
| AA | 2 (0.44) | 0 (0.00) | — | — | — | — |  | 3 (1.67) | 4.10 (0.67-31.32) | 0.125 | 2.84 (0.43-23.19) | 0.276 |
| Dominant model |  |  | 1.26 (0.46-2.93) | 0.615 | 0.90 (0.29-2.39) | 0.837 |  |  | 1.82 (1.11-2.95) | **0.016** | 1.68 (0.99-2.83) | 0.052 |
| Recessive model |  |  | 1.32 (0.48-3.08) | 0.546 | 0.93 (0.30-2.49) | 0.885 |  |  | 1.70 (1.02-2.78) | **0.039** | 1.60 (0.92-2.74) | 0.090 |
| Additive model |  |  | 0.76 (0.32-2.07) | 0.546 | 1.08 (0.40-3.385) | 0.885 |  |  | 0.59 (0.36-0.98) | **0.039** | 0.63 (0.37-1.09) | 0.090 |
| *TRPM8*-rs7577262 G>A | |  |  |  |  |  |  |  |  |  |  |  |
| GG | 169 (37.39) | 24 (52.17) | ref |  | ref |  |  | 82 (45.56) | ref |  | ref |  |
| GA | 221 (48.89) | 15 (32.61) | 0.48 (0.24-0.93) | **0.032** | 0.52 (0.25-1.09) | 0.085 |  | 79 (43.89) | 0.74 (0.51-1.07) | 0.104 | 0.79 (0.53-1.17) | 0.232 |
| AA | 62 (13.72) | 7 (15.22) | 0.80 (0.30-1.85) | 0.614 | 0.49 (0.15-1.35) | 0.190 |  | 19 (10.56) | 0.63 (0.35-1.11) | 0.119 | 0.66 (0.35-1.19) | 0.177 |
| Dominant model |  |  | 0.89 (0.40-2.24) | 0.779 | 1.53 (0.58-4.71) | 0.419 |  |  | 1.35 (0.80-2.38) | 0.285 | 1.35 (0.77-2.46) | 0.315 |
| Recessive model |  |  | 0.51 (0.26-0.95) | **0.038** | 0.62 (0.30-1.23) | 0.178 |  |  | 0.82 (0.58-1.16) | 0.256 | 0.87 (0.59-1.26) | 0.446 |
| Additive model |  |  | 1.98 (1.06-3.86) | **0.038** | 1.63 (0.81-3.36) | 0.178 |  |  | 1.22 (0.87-1.73) | 0.256 | 1.16 (0. 80-1.68) | 0.446 |
| *TRPV1*-rs8065080 C>T | |  |  |  |  |  |  |  |  |  |  |  |
| CC | 158 (34.96) | 22 (47.83) | ref |  | ref |  |  | 65 (36.11) | ref |  | ref |  |
| TC | 216 (47.79) | 18 (39.13) | 0.60 (0.307-1.15) | 0.125 | 0.46 (0.22-0.97) | **0.042** |  | 94 (52.22) | 1.06 (0.73-1.55) | 0.770 | 1.06 (0.71-1.60) | 0.772 |
| TT | 78 (17.26) | 6 (13.04) | 0.55 (0.197-1.34) | 0.217 | 0.45 (0.14-1.26) | 0.149 |  | 21 (11.67) | 0.65 (0.37-1.13) | 0.139 | 0.61 (0.33-1.10) | 0.108 |
| Dominant model |  |  | 1.39 (0.611-3.75) | 0.469 | 1.46 (0.57-4.42) | 0.466 |  |  | 1.58 (0.96-2.70) | 0.083 | 1.71 (0.99-3.04) | 0.060 |
| Recessive model |  |  | 0.70 (0.37-1.30) | 0.264 | 0.57 (0.28-1.14) | 0.118 |  |  | 1.19 (0.85-1.69) | 0.314 | 1.23 (0.85-1.79) | 0.280 |
| Additive model |  |  | 1.42 (0.77-2.69) | 0.264 | 1.75 (0.88-3.58) | 0.118 |  |  | 0.84 (0.59-1.18) | 0.314 | 0.81 (0.56-1.18) | 0.280 |
| *TRPM8*-rs10166942 C>T | |  |  |  |  |  |  |  |  |  |  |  |
| CC | 185 (40.93) | 17 (36.96) | ref |  | ref |  |  | 65 (36.11) | ref |  | ref |  |
| TC | 219 (48.45) | 22 (47.83) | 1.09 (0.57-2.15) | 0.792 | 1.72 (0.81-3.77) | 0.166 |  | 88 (48.89) | 1.14 (0.79-1.67) | 0.484 | 1.18 (0.79-1.78) | 0.417 |
| TT | 48 (10.62) | 7 (15.22) | 1.59 (0.59-3.91) | 0.333 | 2.17 (0.72-6.12) | 0.149 |  | 27 (15.00) | 1.60 (0.92-2.76) | 0.093 | 1.54 (0.85-2.77) | 0.152 |
| Dominant model |  |  | 1.18 (0.64-2.25) | 0.601 | 1.81 (0.89-3.85) | 0.108 |  |  | 1.23 (0.86-1.76) | 0.264 | 1.25 (0.85-1.84) | 0.253 |
| Recessive model |  |  | 0.98 (0.53-1.79) | 0.936 | 1.41 (0.71-2.83) | 0.335 |  |  | 1.02 (0.72-1.44) | 0.921 | 1.06 (0.73-1.54) | 0.755 |
| Additive model |  |  | 1.03 (0.56-1.89) | 0.936 | 0.71 (0.35-1.42) | 0.335 |  |  | 0.98 (0.70-1.39) | 0.921 | 0.94 (0.65-1.37) | 0.755 |
| *TRPM8*-rs17862920 C>T | |  |  |  |  |  |  |  |  |  |  |  |
| CC | 243 (53.76) | 28 (60.87) | ref |  | ref |  |  | 115 (63.89) | ref |  | ref |  |
| CT | 177 (39.16) | 14 (30.44) | 0.69 (0.34-1.32) | 0.271 | 0.63 (0.28-1.33) | 0.235 |  | 58 (32.22) | 0.69 (0.48-1.00) | 0.052 | 0.74 (0.50-1.11) | 0.148 |
| TT | 32 (7.08) | 4 (8.70) | 1.09 (0.31-2.99) | 0.886 | 0.79 (0.18-2.73) | 0.730 |  | 7 (3.89) | 0.46 (0.18-1.02) | 0.074 | 0.48 (0.18-1.13) | 0.114 |
| Dominant model |  |  | 0.80 (0.30-2.78) | 0.687 | 1.07 (0.32-4.61) | 0.919 |  |  | 1.88 (0.86-4.72) | 0.138 | 1.84 (0.80-4.83) | 0.180 |
| Recessive model |  |  | 0.68 (0.34-1.29) | 0.249 | 0.65 (0.30-1.34) | 0.254 |  |  | 0.74 (0.51-1.06) | 0.104 | 0.79 (0.53-1.18) | 0.252 |
| Additive model |  |  | 1.47 (0.78-2.92) | 0.249 | 1.55 (0.75-3.39) | 0.254 |  |  | 1.35 (0.94-1.96) | 0.104 | 1.26 (0.85-1.88) | 0.252 |
| *TRPV1*-rs222741 A>G | |  |  |  |  |  |  |  |  |  |  |  |
| AA | 291 (64.38) | 32 (69.57) | ref |  | ref |  |  | 114 (63.33) | ref |  | ref |  |
| GA | 146 (32.30) | 13 (28.26) | 0.81 (0.40-1.56) | 0.540 | 0.62 (0.28-1.32) | 0.234 |  | 60 (33.33) | 1.05 (0.72-1.52) | 0.800 | 0.94 (0.63-1.41) | 0.771 |
| GG | 15 (3.32) | 1 (2.17) | 0.61 (0.03-3.15) | 0.633 | 0.58 (0.03-3.51) | 0.619 |  | 6 (3.33) | 1.02 (0.36-2.58) | 0.966 | 0.97 (0.32-2.63) | 0.950 |
| Dominant model |  |  | 1.55 (0.30-28.25) | 0.677 | 1.47 (0.25-28.48) | 0.729 |  |  | 1.00 (0.40-2.83) | 0.993 | 1.01 (0.38-3.05) | 0.982 |
| Recessive model |  |  | 0.83 (0.41-1.58) | 0.576 | 0.64 (0.29-1.35) | 0.259 |  |  | 1.05 (0.72-1.51) | 0.803 | 0.94 (0.63-1.40) | 0.775 |
| Additive model |  |  | 1.21 (0.63-2.45) | 0.576 | 1.56 (0.74-3.49) | 0.259 |  |  | 0.95 (0.66-1.38) | 0.803 | 1.06 (0.71-1.59) | 0.775 |
| *SHANK2*-rs3020047 C>T | |  |  |  |  |  |  |  |  |  |  |  |
| CC | 342 (75.66) | 35 (76.09) | ref |  | ref |  |  | 126 (70.00) | ref |  | ref |  |
| TC | 102 (22.57) | 11 (23.91) | 1.05 (0.50-2.09) | 0.885 | 0.96 (0.40-2.12) | 0.921 |  | 50 (27.78) | 1.33 (0.89-1.97) | 0.157 | 1.33 (0.86-2.05) | 0.197 |
| TT | 8 (1.77) | 0 (0.00) | — | — | — | — |  | 4 (2.22) | 1.36 (0.36-4.39) | 0.623 | 1.34 (0.33-4.75) | 0.657 |
| Dominant model |  |  | 0.98 (0.46-1.93) | 0.949 | 0.86 (0.37-1.89) | 0.720 |  |  | 1.33 (0.90-1.95) | 0.143 | 1.33 (0.87-2.02) | 0.181 |
| Recessive model |  |  | 1.08 (0.51-2.14) | 0.835 | 0.99 (0.42-2.18) | 0.980 |  |  | 1.32 (0.89-1.95) | 0.167 | 1.32 (0.85-2.03) | 0.207 |
| Additive model |  |  | 0.93 (0.47-1.97) | 0.835 | 1.01 (0.46-2.40) | 0.980 |  |  | 0.76 (0.51-1.13) | 0.167 | 0.76 (0.49-1.17) | 0.207 |
| *TRPV4*-rs3742037 G>A | |  |  |  |  |  |  |  |  |  |  |  |
| GG | 297 (65.71) | 32 (69.57) | ref |  | ref |  |  | 121 (67.22) | ref |  | ref |  |
| GA | 141 (31.20) | 10 (21.74) | 0.66 (0.30-1.33) | 0.267 | 0.67 (0.28-1.47) | 0.338 |  | 54 (30.00) | 0.94 (0.64-1.37) | 0.749 | 0.89 (0.59-1.33) | 0.561 |
| AA | 14 (3.10) | 4 (8.70) | 2.65 (0.72-7.92) | 0.102 | 1.48 (0.29-6.29) | 0.611 |  | 5 (2.78) | 0.88 (0.28-2.35) | 0.805 | 0.89 (0.27-2.52) | 0.833 |
| Dominant model |  |  | 0.84 (0.42-1.59) | 0.599 | 0.77 (0.35-1.59) | 0.492 |  |  | 0.93 (0.65-1.34) | 0.717 | 0.89 (0.60-1.31) | 0.549 |
| Recessive model |  |  | 0.61 (0.28-1.23) | 0.187 | 0.65 (0.28-1.42) | 0.305 |  |  | 0.95 (0.65-1.37) | 0.769 | 0.89 (0.59-1.33) | 0.575 |
| Additive model |  |  | 1.63 (0.82-3.56) | 0.187 | 1.53 (0.70-3.62) | 0.305 |  |  | 1.06 (0.73-1.55) | 0.769 | 1.12 (0.75-1.69) | 0.575 |
| *SHANK1*-rs3745521 A>G | |  |  |  |  |  |  |  |  |  |  |  |
| AA | 130 (28.76) | 12 (26.09) | ref |  | ref |  |  | 60 (33.33) | ref |  | ref |  |
| AG | 219 (48.45) | 19 (41.30) | 0.94 (0.45-2.05) | 0.872 | 0.89 (0.38-2.12) | 0.778 |  | 83 (46.11) | 0.82 (0.55-1.22) | 0.330 | 0.77 (0.50-1.18) | 0.230 |
| GG | 103 (22.79) | 15 (32.61) | 1.58 (0.71-3.58) | 0.265 | 1.29 (0.50-3.32) | 0.597 |  | 37 (20.56) | 0.78 (0.48-1.26) | 0.311 | 0.68 (0.40-1.15) | 0.154 |
| Dominant model |  |  | 0.61 (0.32-1.20) | 0.139 | 0.72 (0.34-1.57) | 0.392 |  |  | 1.14 (0.75-1.76) | 0.542 | 1.25 (0.79-1.99) | 0.346 |
| Recessive model |  |  | 0.75 (0.40-1.38) | 0.356 | 0.78 (0.39-1.55) | 0.475 |  |  | 0.91 (0.64-1.29) | 0.595 | 0.90 (0.62-1.31) | 0.593 |
| Additive model |  |  | 1.34 (0.73-2.50) | 0.356 | 1.29 (0.65-2.60) | 0.475 |  |  | 1.10 (0.78-1.56) | 0.595 | 1.11 (0.76-1.61) | 0.593 |
| *SHANK2*-rs55678639 A>G | |  |  |  |  |  |  |  |  |  |  |  |
| AA | 284 (62.83) | 26 (56.52) | ref |  | ref |  |  | 124 (68.89) | ref |  | ref |  |
| AG | 146 (32.30) | 17 (36.96) | 1.27 (0.66-2.40) | 0.464 | 1.13 (0.54-2.32) | 0.740 |  | 48 (26.67) | 0.75 (0.51-1.11) | 0.152 | 0.73 (0.48-1.11) | 0.146 |
| GG | 22 (4.87) | 3 (6.52) | 1.49 (0.34-4.68) | 0.539 | 1.12 (0.21-4.24) | 0.877 |  | 8 (4.44) | 0.83 (0.34-1.85) | 0.668 | 0.75 (0.29-1.77) | 0.532 |
| Dominant model |  |  | 0.73 (0.24-3.19) | 0.626 | 0.93 (0.25-4.86) | 0.921 |  |  | 1.10 (0.50-2.68) | 0.822 | 1.21 (0.52-3.08) | 0.671 |
| Recessive model |  |  | 1.23 (0.64-2.29) | 0.522 | 1.12 (0.54-2.26) | 0.756 |  |  | 0.76 (0.52-1.11) | 0.166 | 0.75 (0.49-1.13) | 0.168 |
| Additive model |  |  | 0.81 (0.44-1.56) | 0.522 | 0.89 (0.44-1.87) | 0.756 |  |  | 1.31 (0.90-1.94) | 0.166 | 1.34 (0.89-2.04) | 0.168 |

Bold type indicates *P* < 0.05. Abbreviations: MA, Migraine with aura; MO, Migraine without aura. OR_adj_ (95% CI) adjusted factors: age, sex, BMI, marital status, nationality, educational level, smoking, alcohol, exercise, history of hypertension, history of diabetes, family history of diabetes, family history of migraine, SAS grade, PHQ9 grade, PSQI grade.

**Table S10.** Association signals between genotypes of SNP rs2227283 and gene expression levels of *GRIK2* in multiple types of human tissues.

| Gene Symbol | SNP | *P*-Value | NES | T-statistic | Tissue |
| --- | --- | --- | --- | --- | --- |
| *GRIK2* | rs2227283 | 0.014 | 0.076 | 2.5 | Brain - Nucleus accumbens (basal ganglia) |
| *GRIK2* | rs2227283 | 0.02 | 0.2 | 2.4 | Ovary |
| *GRIK2* | rs2227283 | 0.026 | 0.084 | 2.2 | Heart - Atrial Appendage |
| *GRIK2* | rs2227283 | 0.027 | 0.086 | 2.2 | Brain - Cortex |
| *GRIK2* | rs2227283 | 0.041 | 0.069 | 2 | Stomach |
| *GRIK2* | rs2227283 | 0.061 | 0.14 | 1.9 | Brain - Substantia nigra |
| *GRIK2* | rs2227283 | 0.073 | 0.088 | 1.8 | Testis |
| *GRIK2* | rs2227283 | 0.1 | 0.18 | 1.6 | Uterus |
| *GRIK2* | rs2227283 | 0.13 | -0.073 | -1.5 | Heart - Left Ventricle |
| *GRIK2* | rs2227283 | 0.14 | 0.058 | 1.5 | Artery - Tibial |
| *GRIK2* | rs2227283 | 0.14 | 0.047 | 1.5 | Brain - Hippocampus |
| *GRIK2* | rs2227283 | 0.15 | 0.066 | 1.5 | Skin - Not Sun Exposed (Suprapubic) |
| *GRIK2* | rs2227283 | 0.16 | -0.07 | -1.4 | Adrenal Gland |
| *GRIK2* | rs2227283 | 0.16 | 0.055 | 1.4 | Thyroid |
| *GRIK2* | rs2227283 | 0.21 | 0.053 | 1.3 | Brain - Cerebellum |
| *GRIK2* | rs2227283 | 0.26 | -0.093 | -1.1 | Minor Salivary Gland |
| *GRIK2* | rs2227283 | 0.26 | -0.077 | -1.1 | Vagina |
| *GRIK2* | rs2227283 | 0.27 | 0.052 | 1.1 | Breast - Mammary Tissue |
| *GRIK2* | rs2227283 | 0.28 | -0.064 | -1.1 | Artery - Aorta |
| *GRIK2* | rs2227283 | 0.3 | -0.035 | -1 | Colon - Transverse |
| *GRIK2* | rs2227283 | 0.33 | 0.028 | 0.97 | Nerve - Tibial |
| *GRIK2* | rs2227283 | 0.35 | -0.034 | -0.93 | Esophagus - Gastroesophageal Junction |
| *GRIK2* | rs2227283 | 0.36 | -0.032 | -0.93 | Brain - Caudate (basal ganglia) |
| *GRIK2* | rs2227283 | 0.39 | 0.057 | 0.86 | Pancreas |
| *GRIK2* | rs2227283 | 0.49 | -0.03 | -0.7 | Brain - Hypothalamus |
| *GRIK2* | rs2227283 | 0.52 | 0.017 | 0.65 | Esophagus - Muscularis |
| *GRIK2* | rs2227283 | 0.53 | 0.03 | 0.63 | Lung |
| *GRIK2* | rs2227283 | 0.54 | 0.026 | 0.61 | Esophagus - Mucosa |
| *GRIK2* | rs2227283 | 0.57 | -0.018 | -0.57 | Muscle - Skeletal |
| *GRIK2* | rs2227283 | 0.58 | 0.037 | 0.56 | Brain - Amygdala |
| *GRIK2* | rs2227283 | 0.64 | 0.02 | 0.47 | Cells - Cultured fibroblasts |
| *GRIK2* | rs2227283 | 0.65 | 0.019 | 0.46 | Brain - Frontal Cortex (BA9) |
| *GRIK2* | rs2227283 | 0.67 | -0.019 | -0.43 | Brain - Anterior cingulate cortex (BA24) |
| *GRIK2* | rs2227283 | 0.7 | 0.021 | 0.38 | Brain - Spinal cord (cervical c-1) |
| *GRIK2* | rs2227283 | 0.71 | -0.029 | -0.37 | Spleen |
| *GRIK2* | rs2227283 | 0.76 | -0.012 | -0.31 | Skin - Sun Exposed (Lower leg) |
| *GRIK2* | rs2227283 | 0.78 | -0.0091 | -0.28 | Adipose - Subcutaneous |
| *GRIK2* | rs2227283 | 0.78 | 0.013 | 0.28 | Pituitary |
| *GRIK2* | rs2227283 | 0.79 | -0.0099 | -0.27 | Colon - Sigmoid |
| *GRIK2* | rs2227283 | 0.8 | 0.015 | 0.26 | Brain - Cerebellar Hemisphere |
| *GRIK2* | rs2227283 | 0.81 | 0.027 | 0.24 | Kidney - Cortex |
| *GRIK2* | rs2227283 | 0.82 | -0.015 | -0.23 | Prostate |
| *GRIK2* | rs2227283 | 0.85 | -0.0068 | -0.2 | Brain - Putamen (basal ganglia) |
| *GRIK2* | rs2227283 | 0.86 | 0.0094 | 0.17 | Small Intestine - Terminal Ileum |
| *GRIK2* | rs2227283 | 0.9 | 0.0059 | 0.13 | Adipose - Visceral (Omentum) |
| *GRIK2* | rs2227283 | 0.9 | -0.0079 | -0.13 | Artery - Coronary |

Bold type indicates *P* < 0.001 (0.05/46). NES: normalized effect size.

**Table S11.** Association signals between genotypes of SNP rs8065080 and gene expression levels of *TRPV1* in multiple types of human tissues.

| Gene Symbol | SNP | *P*-Value | NES | T-statistic | Tissue |
| --- | --- | --- | --- | --- | --- |
| *TRPV1* | rs8065080 | **2.30E-05** | -0.13 | -4.3 | Skin - Sun Exposed (Lower leg) |
| *TRPV1* | rs8065080 | **5.20E-05** | -0.13 | -4.1 | Skin - Not Sun Exposed (Suprapubic) |
| *TRPV1* | rs8065080 | **9.00E-05** | -0.27 | -4.1 | Vagina |
| *TRPV1* | rs8065080 | **9.50E-05** | -0.15 | -4 | Artery - Aorta |
| *TRPV1* | rs8065080 | 0.0014 | -0.1 | -3.2 | Whole Blood |
| *TRPV1* | rs8065080 | 0.002 | -0.078 | -3.1 | Cells - Cultured fibroblasts |
| *TRPV1* | rs8065080 | 0.0023 | -0.13 | -3.1 | Testis |
| *TRPV1* | rs8065080 | 0.011 | -0.085 | -2.6 | Esophagus - Gastroesophageal Junction |
| *TRPV1* | rs8065080 | 0.016 | 0.13 | 2.4 | Artery - Coronary |
| *TRPV1* | rs8065080 | 0.016 | -0.073 | -2.4 | Thyroid |
| *TRPV1* | rs8065080 | 0.02 | 0.13 | 2.4 | Brain - Nucleus accumbens (basal ganglia) |
| *TRPV1* | rs8065080 | 0.021 | -0.072 | -2.3 | Colon - Transverse |
| *TRPV1* | rs8065080 | 0.033 | -0.1 | -2.1 | Pancreas |
| *TRPV1* | rs8065080 | 0.04 | 0.11 | 2.1 | Pituitary |
| *TRPV1* | rs8065080 | 0.042 | -0.14 | -2 | Spleen |
| *TRPV1* | rs8065080 | 0.044 | -0.059 | -2 | Esophagus - Muscularis |
| *TRPV1* | rs8065080 | 0.047 | -0.12 | -2 | Ovary |
| *TRPV1* | rs8065080 | 0.065 | -0.071 | -1.9 | Lung |
| *TRPV1* | rs8065080 | 0.065 | -0.14 | -1.9 | Minor Salivary Gland |
| *TRPV1* | rs8065080 | 0.067 | 0.054 | 1.8 | Artery - Tibial |
| *TRPV1* | rs8065080 | 0.074 | -0.057 | -1.8 | Adipose - Visceral (Omentum) |
| *TRPV1* | rs8065080 | 0.12 | -0.06 | -1.5 | Esophagus - Mucosa |
| *TRPV1* | rs8065080 | 0.13 | 0.13 | 1.5 | Brain - Substantia nigra |
| *TRPV1* | rs8065080 | 0.13 | -0.19 | -1.5 | Cells - EBV-transformed lymphocytes |
| *TRPV1* | rs8065080 | 0.14 | -0.095 | -1.5 | Uterus |
| *TRPV1* | rs8065080 | 0.16 | -0.044 | -1.4 | Muscle - Skeletal |
| *TRPV1* | rs8065080 | 0.17 | 0.062 | 1.4 | Brain - Cortex |
| *TRPV1* | rs8065080 | 0.29 | 0.042 | 1.1 | Heart - Atrial Appendage |
| *TRPV1* | rs8065080 | 0.35 | 0.049 | 0.95 | Brain - Caudate (basal ganglia) |
| *TRPV1* | rs8065080 | 0.35 | 0.057 | 0.93 | Brain - Cerebellar Hemisphere |
| *TRPV1* | rs8065080 | 0.37 | -0.049 | -0.9 | Brain - Frontal Cortex (BA9) |
| *TRPV1* | rs8065080 | 0.37 | -0.039 | -0.89 | Small Intestine - Terminal Ileum |
| *TRPV1* | rs8065080 | 0.38 | 0.051 | 0.88 | Brain - Anterior cingulate cortex (BA24) |
| *TRPV1* | rs8065080 | 0.41 | 0.056 | 0.83 | Brain - Amygdala |
| *TRPV1* | rs8065080 | 0.46 | 0.041 | 0.74 | Brain - Hippocampus |
| *TRPV1* | rs8065080 | 0.47 | 0.047 | 0.73 | Brain - Hypothalamus |
| *TRPV1* | rs8065080 | 0.52 | -0.023 | -0.64 | Liver |
| *TRPV1* | rs8065080 | 0.55 | -0.024 | -0.6 | Breast - Mammary Tissue |
| *TRPV1* | rs8065080 | 0.56 | -0.026 | -0.58 | Stomach |
| *TRPV1* | rs8065080 | 0.59 | -0.031 | -0.54 | Brain - Cerebellum |
| *TRPV1* | rs8065080 | 0.61 | 0.03 | 0.51 | Brain - Putamen (basal ganglia) |
| *TRPV1* | rs8065080 | 0.74 | 0.012 | 0.33 | Heart - Left Ventricle |
| *TRPV1* | rs8065080 | 0.77 | -0.0087 | -0.29 | Adipose - Subcutaneous |
| *TRPV1* | rs8065080 | 0.77 | -0.017 | -0.29 | Adrenal Gland |
| *TRPV1* | rs8065080 | 0.79 | 0.0096 | 0.27 | Colon - Sigmoid |
| *TRPV1* | rs8065080 | 0.8 | 0.013 | 0.25 | Prostate |
| *TRPV1* | rs8065080 | 0.85 | -0.0052 | -0.19 | Nerve - Tibial |
| *TRPV1* | rs8065080 | 0.88 | 0.013 | 0.16 | Kidney - Cortex |
| *TRPV1* | rs8065080 | 0.94 | 0.0058 | 0.078 | Brain - Spinal cord (cervical c-1) |

Bold type indicates *P* < 0.001 (0.05/49). NES: normalized effect size.

**Table S12.** Association signals between genotypes of SNP rs7217270 and gene expression levels of *TRPV3* in multiple types of human tissues.

| Gene Symbol | SNP | *P*-Value | NES | T-statistic | Tissue |
| --- | --- | --- | --- | --- | --- |
| *TRPV3* | rs7217270 | **0.00084** | 0.26 | 3.4 | Adrenal Gland |
| *TRPV3* | rs7217270 | 0.0026 | -0.19 | -3.1 | Brain - Putamen (basal ganglia) |
| *TRPV3* | rs7217270 | 0.0042 | -0.061 | -2.9 | Skin - Sun Exposed (Lower leg) |
| *TRPV3* | rs7217270 | 0.0054 | 0.23 | 2.8 | Pancreas |
| *TRPV3* | rs7217270 | 0.0063 | -0.2 | -2.8 | Brain - Substantia nigra |
| *TRPV3* | rs7217270 | 0.0069 | -0.22 | -2.7 | Ovary |
| *TRPV3* | rs7217270 | 0.007 | -0.2 | -2.7 | Spleen |
| *TRPV3* | rs7217270 | 0.014 | -0.17 | -2.5 | Brain - Caudate (basal ganglia) |
| *TRPV3* | rs7217270 | 0.019 | -0.085 | -2.4 | Nerve - Tibial |
| *TRPV3* | rs7217270 | 0.024 | -0.19 | -2.3 | Brain - Cerebellum |
| *TRPV3* | rs7217270 | 0.024 | -0.093 | -2.3 | Esophagus - Gastroesophageal Junction |
| *TRPV3* | rs7217270 | 0.034 | -0.062 | -2.1 | Whole Blood |
| *TRPV3* | rs7217270 | 0.065 | -0.097 | -1.9 | Artery - Aorta |
| *TRPV3* | rs7217270 | 0.077 | -0.078 | -1.8 | Esophagus - Mucosa |
| *TRPV3* | rs7217270 | 0.081 | -0.15 | -1.8 | Brain - Amygdala |
| *TRPV3* | rs7217270 | 0.086 | -0.073 | -1.7 | Thyroid |
| *TRPV3* | rs7217270 | 0.09 | -0.12 | -1.7 | Brain - Frontal Cortex (BA9) |
| *TRPV3* | rs7217270 | 0.1 | -0.092 | -1.6 | Brain - Cortex |
| *TRPV3* | rs7217270 | 0.11 | -0.062 | -1.6 | Artery - Tibial |
| *TRPV3* | rs7217270 | 0.12 | -0.12 | -1.6 | Brain - Anterior cingulate cortex (BA24) |
| *TRPV3* | rs7217270 | 0.13 | -0.14 | -1.5 | Brain - Cerebellar Hemisphere |
| *TRPV3* | rs7217270 | 0.13 | -0.08 | -1.5 | Brain - Hippocampus |
| *TRPV3* | rs7217270 | 0.13 | -0.12 | -1.5 | Brain - Spinal cord (cervical c-1) |
| *TRPV3* | rs7217270 | 0.19 | -0.15 | -1.3 | Liver |
| *TRPV3* | rs7217270 | 0.21 | -0.046 | -1.2 | Adipose - Subcutaneous |
| *TRPV3* | rs7217270 | 0.23 | -0.056 | -1.2 | Lung |
| *TRPV3* | rs7217270 | 0.27 | -0.057 | -1.1 | Heart - Atrial Appendage |
| *TRPV3* | rs7217270 | 0.27 | -0.11 | -1.1 | Vagina |
| *TRPV3* | rs7217270 | 0.29 | -0.042 | -1.1 | Adipose - Visceral (Omentum) |
| *TRPV3* | rs7217270 | 0.3 | -0.036 | -1 | Esophagus - Muscularis |
| *TRPV3* | rs7217270 | 0.31 | -0.072 | -1 | Small Intestine - Terminal Ileum |
| *TRPV3* | rs7217270 | 0.33 | -0.12 | -0.97 | Uterus |
| *TRPV3* | rs7217270 | 0.37 | -0.039 | -0.91 | Breast - Mammary Tissue |
| *TRPV3* | rs7217270 | 0.38 | 0.071 | 0.87 | Pituitary |
| *TRPV3* | rs7217270 | 0.38 | -0.06 | -0.88 | Prostate |
| *TRPV3* | rs7217270 | 0.39 | -0.034 | -0.87 | Cells - Cultured fibroblasts |
| *TRPV3* | rs7217270 | 0.43 | -0.055 | -0.79 | Brain - Nucleus accumbens (basal ganglia) |
| *TRPV3* | rs7217270 | 0.54 | -0.058 | -0.61 | Minor Salivary Gland |
| *TRPV3* | rs7217270 | 0.55 | -0.032 | -0.6 | Testis |
| *TRPV3* | rs7217270 | 0.58 | -0.028 | -0.56 | Heart - Left Ventricle |
| *TRPV3* | rs7217270 | 0.58 | -0.082 | -0.56 | Kidney - Cortex |
| *TRPV3* | rs7217270 | 0.59 | -0.017 | -0.55 | Skin - Not Sun Exposed (Suprapubic) |
| *TRPV3* | rs7217270 | 0.66 | -0.021 | -0.44 | Artery - Coronary |
| *TRPV3* | rs7217270 | 0.71 | -0.025 | -0.38 | Brain - Hypothalamus |
| *TRPV3* | rs7217270 | 0.72 | -0.019 | -0.36 | Stomach |
| *TRPV3* | rs7217270 | 0.74 | -0.014 | -0.33 | Muscle - Skeletal |
| *TRPV3* | rs7217270 | 0.82 | -0.013 | -0.23 | Colon - Transverse |
| *TRPV3* | rs7217270 | 0.9 | 0.012 | 0.12 | Cells - EBV-transformed lymphocytes |
| *TRPV3* | rs7217270 | 0.98 | 0.00096 | 0.021 | Colon - Sigmoid |

Bold type indicates *P* < 0.001 (0.05/49). NES: normalized effect size.

**Table S13.** Association signals between genotypes of SNP rs7577262 and gene expression levels of *TRPM8* in multiple types of human tissues.

| Gene Symbol | SNP | *P*-Value | NES | T-statistic | Tissue |
| --- | --- | --- | --- | --- | --- |
| *TRPM8* | rs7577262 | 0.056 | 0.11 | 1.9 | Prostate |
| *TRPM8* | rs7577262 | 0.11 | 0.14 | 1.6 | Testis |
| *TRPM8* | rs7577262 | 0.13 | -0.16 | -1.5 | Pituitary |
| *TRPM8* | rs7577262 | 0.27 | -0.18 | -1.1 | Kidney - Cortex |
| *TRPM8* | rs7577262 | 0.61 | 0.073 | 0.51 | Brain - Hypothalamus |
| *TRPM8* | rs7577262 | 0.67 | -0.048 | -0.43 | Brain - Caudate (basal ganglia) |
| *TRPM8* | rs7577262 | 0.67 | 0.034 | 0.43 | Liver |
| *TRPM8* | rs7577262 | 0.73 | 0.039 | 0.34 | Brain - Putamen (basal ganglia) |
| *TRPM8* | rs7577262 | 0.87 | 0.025 | 0.17 | Brain - Cerebellum |
| *TRPM8* | rs7577262 | 0.94 | -0.0094 | -0.07 | Brain - Cerebellar Hemisphere |

Bold type indicates *P* < 0.005 (0.05/10). NES: normalized effect size.

**Table S14.** **ESE or ESS for *SLC17A8* rs11110359 and *TRPV1* rs8065080.**

| SNP | Allele | Position | Prediction Strand | Forward Sequence | Matrix | Score | Method |
| --- | --- | --- | --- | --- | --- | --- | --- |
| rs11110359 | G | 4 | + | GACGTC | SRp55 | 2.73 | ESEfind |
|  | G | 6 | + | CAGACGT | SF2ASF2 | 4.8 | ESEfind |
|  | A | 6 | + | CAGACAT | SF2ASF2 | 3.02 | ESEfind |
|  | G | 6 | + | CAGACGT | SF2ASF1 | 5.99 | ESEfind |
|  | A | 6 | + | CAGACAT | SF2ASF1 | 3.42 | ESEfind |
|  | A | 5 | + | AGACAT | NA | NA | RESCUE-ESE |
| rs8065080 | T | 1 | + | TGTAGA | SRp55 | 2.8 | ESEfind |
|  | C | 6 | + | GACGAC | NA | NA | RESCUE-ESE |

ESE: Exonic Splicing Enhancer; ESS: Exonic Splicing Silencer.

**Table S15.** **MicroRNA-binding sites for *SLC17A8* rs11568537 and *SHANK2* rs55678639.**

| SNP | Allele | Position | Prediction Strand | Forward Sequence | miRNA | Score | Energy |
| --- | --- | --- | --- | --- | --- | --- | --- |
| rs55678639 | A | 15 | - | AAATAAACTATGTTATCAGCTCTT | hsa-miR-495 | 141 | -9.59 |
|  | G | 15 | - | AAATAAACTATGTTGTCAGCTCTT | hsa-miR-495 | 145 | -9.84 |
|  | A | 17 | - | AAAAATAAACTATGTTATCAGCt | hsa-miR-548b-5p | 153 | -12.51 |
|  | G | 17 | - | AAAAATAAACTATGTTGTCAGCt | hsa-miR-548b-5p | 153 | -11.83 |
|  | A | 17 | - | AAAAATAAACTATGTTATCAGCt | hsa-miR-548c-5p | 141 | -7.76 |
|  | G | 17 | - | AAAAATAAACTATGTTGTCAGCt | hsa-miR-548c-5p | 141 | -7.51 |
|  | A | 17 | - | AAAAATAAACTATGTTATCAGCt | hsa-miR-548d-5p | 149 | -10.24 |
|  | G | 17 | - | AAAAATAAACTATGTTGTCAGCt | hsa-miR-548d-5p | 149 | -10.24 |
|  | A | 12 | - | tAAACTATGTTATCAGCTCTTTAg | hsa-miR-548g | 140 | -8.63 |
|  | G | 17 | - | AAAAATAAACTATGTTGTCAGC | hsa-miR-548h | 143 | -7.86 |
|  | A | 17 | - | AAAAATAAACTATGTTATCAGCTCTTTAGT | hsa-miR-548j | 140 | -13.4 |
|  | G | 17 | - | AAAAATAAACTATGTTGTCAGCTCTTTAGT | hsa-miR-548j | 144 | -14.64 |
|  | A | 17 | - | aAAAATAAACTATGttatcagc | hsa-miR-559 | 140 | -7.95 |
|  | G | 17 | - | aAAAATAAACTATGttgtcagc | hsa-miR-559 | 140 | -7.95 |
| rs11568537 | A | 2 | + | aacagaaagtatCCATACCTAt | hsa-miR-331-5p | 150 | -13.47 |
|  | C | 2 | + | accagaaagtatCCATACCTAt | hsa-miR-331-5p | 150 | -15.53 |
